# Mechanistic Insights into Biological Activities of Polyphenolic Compounds from Rosemary Obtained by Inverse Molecular Docking

**DOI:** 10.3390/foods11010067

**Published:** 2021-12-28

**Authors:** Samo Lešnik, Urban Bren

**Affiliations:** 1 Laboratory of Physical Chemistry and Chemical Thermodynamics, Faculty of Chemistry and Chemical Engineering, University of Maribor, Smetanova 17, SI-2000 Maribor, Slovenia; samo.lesnik@um.si; 2 Faculty of Mathematics, Natural Sciences and Information Technologies, University of Primorska, Glagoljaška 8, SI-6000 Koper, Slovenia

**Keywords:** rosemary, inverse molecular docking, carnosol, carnosic acid, rosmanol, rosmarinic acid

## Abstract

Rosemary (*Rosmarinus officinalis* L.) represents a medicinal plant known for its various health-promoting properties. Its extracts and essential oils exhibit antioxidative, anti-inflammatory, anticarcinogenic, and antimicrobial activities. The main compounds responsible for these effects are the diterpenes carnosic acid, carnosol, and rosmanol, as well as the phenolic acid ester rosmarinic acid. However, surprisingly little is known about the molecular mechanisms responsible for the pharmacological activities of rosemary and its compounds. To discern these mechanisms, we performed a large-scale inverse molecular docking study to identify their potential protein targets. Listed compounds were separately docked into predicted binding sites of all non-redundant holo proteins from the Protein Data Bank and those with the top scores were further examined. We focused on proteins directly related to human health, including human and mammalian proteins as well as proteins from pathogenic bacteria, viruses, and parasites. The observed interactions of rosemary compounds indeed confirm the beforementioned activities, whereas we also identified their potential for anticoagulant and antiparasitic actions. The obtained results were carefully checked against the existing experimental findings from the scientific literature as well as further validated using both redocking procedures and retrospective metrics.

## 1. Introduction

Rosemary (*Rosmarinus officinalis* L.), which belongs to the Lamiaceae family, represents an evergreen, perennial, branched shrub that can grow up to three feet tall. It grows fragrant, needle-like, dark green leaves with curved margins and tiny white, pink, purple, or blue flowers [1,2]. The plant is native to the Mediterranean region and its leaves are used extensively in Mediterranean cuisine, mainly as a spice.

Rosemary has been found to possess several bioactive compounds that exert various pharmacological activities, particularly antioxidative [3], anti-inflammatory [4], antidiabetic [5], and antibacterial [6], effects. Moreover, rosemary extracts exhibit promising anticarcinogenic activities in several in vitro [7,8,9] as well as in vivo studies [10,11].

**Figure 1:**
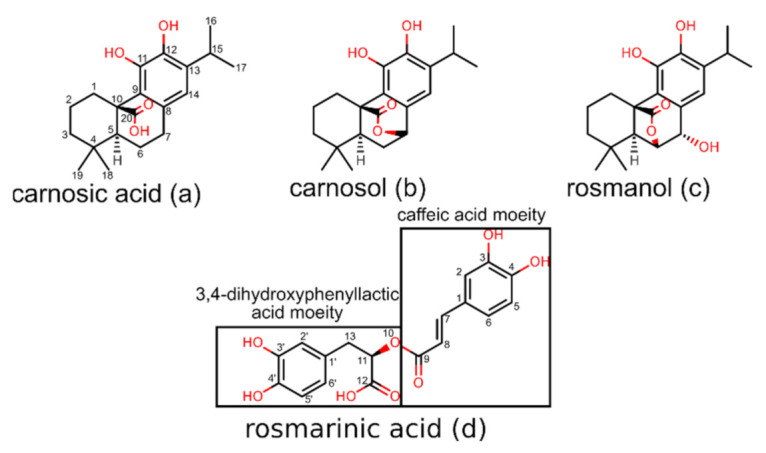
Molecular structures and atom numbering of compounds investigated in the inverse docking study (**a**) molecular structure of carnosic acid, (**b**) carnosol, (**c**) rosmanol, and (**d**) rosmarinic acid.

Carnosic acid (Figure 1a), carnosol (Figure 1b), rosmanol (Figure 1c), and rosmarinic acid (Figure 1d) are most frequently cited in relation to the beneficial pharmacological activities of compounds found in rosemary [12]. Carnosol, carnosic acid, and rosmanol represent polyphenolic diterpenes with similar structures. They consist of the main abietane scaffold, a fused six-membered tricyclic ring system, with one of these rings being aromatic. Carnosic acid represents the major constituent of rosemary and constitutes up to 4% of the dried leaves [13]. However, it is not very stable and, once isolated, undergoes oxidation leading to the formation of the γ-lactone carnosol, which causes it to lose the acidic properties [14]. Oxidation of carnosic acid can alternatively lead to rosmanol, which differs from carnosol in that it has a free hydroxyl group at the C-7 atom and that the γ-lactone is formed via C-20 and C-6 atoms. The three diterpenes form a very effective oxidation cascade, which is vital for the rosemary’s potent antioxidative activity. When carnosic acid is oxidized by free radicals, it forms a quinone derivative. This substance can then undergo isomerization, producing carnosol, or a redox reaction, yielding rosmanol. Thus, carnosic acid, while itself a potent antioxidant, can form two additional substances that also exhibit potent antioxidative activities. This mechanism probably represents the main reason behind the extraordinary antioxidative properties of rosemary [15]. Moreover, these compounds also exhibit antibacterial [16], antiviral [17,18], anti-inflammatory [19,20], antiproliferative [7,8,21,22,23,24,25,26,27,28], and antidepressant [29,30] effects. The study by Romo Vaquero, et al. [31] in rats showed that after oral intake, the glucuronide derivatives of these compounds can be found in plasma as early as 25 min after administration, indicating a good bioavailability. Moreover, carnosic acid was also found in the brain tissue of rats, suggesting that it is able to cross the blood–brain barrier, giving credence to a number of studies in which various positive neuroprotective and cognitive effects were established [32,33].

Rosmarinic acid represents an ester of caffeic acid and 3,4-dihydroxyphenyllactic acid [34]. The structure contains two electroactive catechol moieties that can neutralize free radicals through the electron/proton donor mechanism. Examination of the steps reveals that rosmarinic acid is first oxidized at the caffeic acid moiety of the molecule, while the second step corresponds to the oxidation of the 3,4-dihydroxyphenylic acid moiety. Moreover, the hydroxyl and carboxylic oxygens form a system that exerts good metal chelating properties [35]. Rosmarinic acid can also insert itself into lipid membranes where it effectively inhibits lipid peroxidation [36]. Numerous studies describe that rosmarinic acid exhibits also anti-inflammatory [37,38], antimicrobial [39], anticarcinogenic [7,40,41], and neuroprotective effects [42]. However, unlike the diterpenes in rosemary, the oral bioavailability of rosmarinic acid is poor and amounts to only about 1% in rats [43]. This highlights the need to develop novel delivery systems, such as nanoparticles, to improve the poor pharmacokinetic properties of rosmarinic acid [44,45].

Our aim is to identify potential protein targets of carnosic acid, carnosol, rosmanol, and rosmarinic acid using the inverse docking methodology [46], in which a ligand is docked to a multitude of protein binding sites. The method is typically applied to discover new potential protein targets for small molecule drugs [47] or natural products [48,49,50] and to explain their mechanisms of action in various diseases. To the best of our knowledge such an investigation has never been performed for the major rosemary compounds.

## 2. Materials and Methods

### 2.1. Starting Coordinates of Rosemary Compounds

The initial coordinates of carnosic acid, carnosol, rosmanol, and rosmarinic acid were obtained from the ZINC15 database [51], using ZINC IDs ZINC000003984016, ZINC000003871891, ZINC000031157853, and ZINC0000899870, respectively. Prior to performing inverse molecular docking, all molecules were subjected to a quantum mechanical geometry optimization procedure using the MP2/6-31G* level of theory/basis set combination. This optimization was performed in Gaussian 16 [52].

### 2.2. In Silico Determination of ADME Properties

In silico determined ADME/Tox profiles provide a useful tool for predicting the pharmacological and toxicological properties of investigated molecules [53]. To provide a more detailed prediction of the pharmacokinetic properties of carnosic acid, carnosol, rosmanol, and rosmarinic acid, which would complement the known experimental data, we implemented the SwissADME web server [54]. SwissADME represents a freely available tool that enables robust predictions of absorption, distribution, metabolism, and extraction, based on the two-dimensional data of the molecule. In addition, it yields predictions on drug-likeness based on well-established metrics.

All compounds were inputted on the SwissADME webpage (http://www.swissadme.ch/ date accessed: 20 December 2021) using the Simplified Molecular-Input Line-Entry System (SMILES) strings.

### 2.3. Inverse Molecular Docking

Our goal was to gain mechanistic insight into the potential mechanism of pharmacological actions of the investigated rosemary compounds using CANDOCK (Chemical Atomic Network based Docking) [55] inverse molecular docking on more than 65,000 protein structures potentially associated with human pathologies. Protein binding sites for small molecules were obtained from the ProBiS-Dock Database [56]. The main advantage of defining binding sites in this way is that multiple spherical centroids are defined in advance to describe a very accurate 3D shape that can be used in conjunction with the CANDOCK algorithm. Moreover, binding sites at the interface of multiple protein chains are also considered for docking.

For docking, the CANDOCK algorithm applies a hierarchical approach to reconstruct small molecules from the atomic lattice using graph theory, while applying a generalized statistical potential function for scoring. The docking scores represent approximations of the relative binding free energies and are expressed in arbitrary units. Specifically, CANDOCK finds the best-docked poses of small-molecule fragments and applies a fast-maximum-clique algorithm [57] to link them together. In the molecular reconstruction, the algorithm uses iterative dynamics for better placement of the ligand in the binding pocket. After the initial docking and reconnection procedure is completed, a minimization procedure based on the Chemistry at Harvard Macromolecular Mechanics (CHARMM) force field [58] is performed to model the induced fit of the ligand binding to the protein binding site.

### 2.4. Method Validation

To retrospectively validate our inverse molecular docking procedure, we applied receiver operating characteristic curves (ROC) [59], enrichment curves [60], and predictiveness curves (PC) [61]. Briefly, the ROC metric plot shows a correlation between the true-positive fraction (TPF) on the *y*-axis and the false-positive fraction (FPF) on the *x*-axis. In our case, the TPF represents experimentally confirmed protein targets of rosmarinic acid from the ChEMBL database [62] with the corresponding PDB entries, while the FPF represents all other protein targets from the ProBiS-Dock database. We did not perform an analogous validation for diterpenes as only a small number of confirmed targets is available for them. The area under the ROC curve (ROC AUC) represents a simple measure to evaluate the overall performance of the inverse molecular docking method. The larger the ROC AUC, the more effective is the method at discriminating true from false targets. The enrichment curve represents the early quantification of target proteins from the TPF. Moreover, PC also provides the early detection quantification of target proteins from the TPF, but in addition, it can be used to define the threshold for potential targets from the inverse molecular docking to be tested experimentally. Contrary to ROC, PC can describe the dispersion of the inverse docking scores well. To quantify the early detection, we applied the enrichment factor of 1% of the compounds tested (EF) [63], the Boltzmann-enhanced discrimination of ROC (BEDROC) [59], and the robust initial enrichment (RIE) [63] measures as well. Using PC, the standardized total gain (TG) [61] was also determined, which summarizes the contribution of the inverse molecular docking scores in explaining the probability of targets over the entire protein dataset. To calculate all of the listed measures, the Screening Explorer web server [64] was implemented.

## 3. Results

### 3.1. Inverse Molecular Docking of Diterpenes

**Figure 2:**
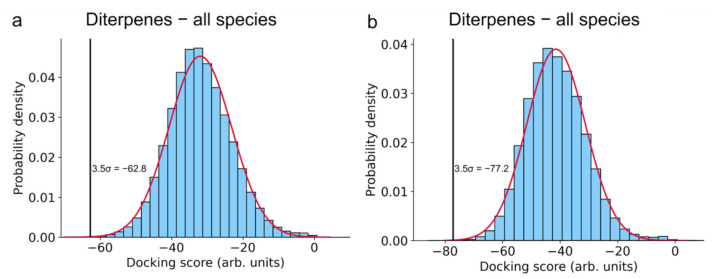
Normal distribution fit of the inverse docking scores. (**a**) Combined distribution of docking scores obtained by inversely docking the rosemary diterpenes carnosic acid, carnosol, and rosmanol to the whole ProBiS-Dock Database. (**b**) Distribution of docking scores for rosmarinic acid. A cut-off criterion of 3.5 σ was used to select the most promising protein–ligand complexes to be further investigated in more detail.

Because of their similar structure and good agreement, the docking results for carnosic acid, carnosol, and rosmanol were combined and analyzed together: the diterpene ligand with the best score for the individual protein was considered. The 0.05% (3.5σ) top scoring proteins from the entire docked database were selected (Figure 2) and among them, those with implications for human health were chosen. Human and mammalian proteins as well as proteins from pathogenic bacteria, viruses, and parasites were considered. Moreover, mammalian proteins were considered in order to increase the protein space available for docking, where we assumed that within the class of mammals, analogous proteins and their binding sites are similar enough so that our findings from non-human mammals are transferable to human proteins.

In Table 1, we present the highest-scoring protein–ligand complexes based on the cut-off criterion of 3.5 σ. Moreover, where data were available, we redocked ligands/drugs that are known to bind to the presented targets using an analogous procedure as the one applied for inverse docking. These results, presented in Table S1, show that in all cases except for K-Ras G12C and enhanced intracellular survival protein, the docking scores of the known ligand/drugs are worse than the ones of the rosemary diterpenes. This indicates an already strong binding affinity of the rosemary compounds, although they have not yet been rationally optimized for these specific protein targets.

#### 3.1.1. K-Ras

**Figure 3:**
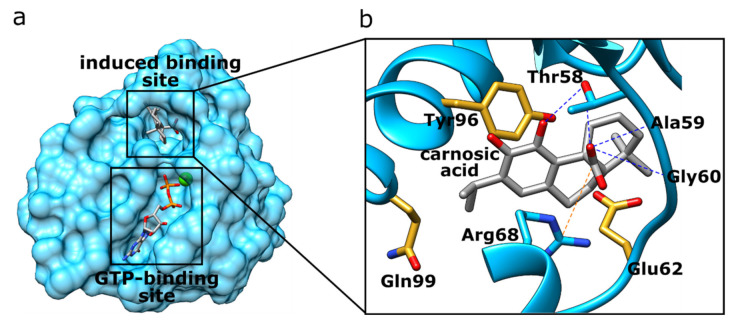
(**a**) K-Ras protein structure highlighting the GTP- and induced-binding site. (**b**) The induced binding site of the K-Ras protein (blue) with docked carnosic acid (carbon atoms depicted in grey). Orange dotted lines represent salt–bridge interactions, and blue dotted lines H-bonding interactions. Amino acid residues forming hydrophobic interactions are denoted with yellow sticks.

K-Ras is a GTPase responsible for relaying signals from outside the cell to the nucleus. It represents a part of the rat sarcoma/mitogen-activated protein kinase (RAS/MAPK) pathway, and K-Ras signaling leads to cell growth, proliferation, and differentiation. K-Ras is of utmost clinical importance as it represents the most frequently mutated oncogene in pancreatic, colon, and lung cancers [72]. Numerous attempts have been made to develop compounds that inhibit the function of K-Ras, but with limited success only [73]. The non-druggability of K-Ras is mainly due to the lack of a well-defined binding pocket, as well as the high affinity for guanosine triphosphate (GTP), with which alternative drug molecules have difficulty competing. Nevertheless, progress has been made in recent years in modulating K-Ras with small-molecule ligands. Fell, et al. [74] developed a potent inhibitor of the oncogenic K-Ras G12C mutant that induces the formation of a new binding pocket near the nucleotide (GTP) binding site (Figure 3a). Binding to this new pocket results in signal inhibition by arresting the enzyme in its inactive state. Interestingly, this induced binding pocket was ranked most favorable of all the protein binding sites tested by our method for carnosic acid (Table 1). Carnosic acid docks at this induced binding site where it forms two hydrogen bonds with Thr58 side chain and two hydrogen bonds to the backbone atoms of Ala59 and Gly60 (Figure 3b, Table S4). A strong salt bridge with a distance of 4.1 Å is additionally created between the carboxylate of carnosic acid and Arg68. Finally, the relatively large hydrophobic ring system of carnosic acid forms hydrophobic interactions with Glu62, Tyr96, and Gln99. Although none of the diterpenes have been previously reported to bind directly to K-Ras, rosemary extracts have indeed been shown to lead to the down-regulation of K-Ras expression in colon cancer cells [75]. This suggests an interesting potential of carnosic acid for a two-pronged attack on the protein by down-regulating its expression and by inhibiting it directly.

#### 3.1.2. Glucosamine/Fructose-6-Phosphate Aminotransferase

In humans, infection with pathogenic strains of *Escherichia coli* leads to various diseases such as gastroenteritis, septic shock, and urinary tract infections. In addition, some strains have been linked to colon cancer because they can synthesize substances that damage DNA [76]. While most *Escherichia coli* infections can be treated with existing antibiotics, such as fluoroquinolones, the proliferation of multidrug-resistant strains produces the need to identify new compounds with antimicrobial activity. Although specific binding of rosemary diterpenes to glucosamine/fructose-6-phosphate aminotransferase (GlmS) is not reported in the scientific literature, a number of studies shows that rosemary compounds indeed exhibit activity against *Escherichia coli* [66,67,68]. Since no mechanism of this inhibition has yet been reported, we speculate that carnosic acid may bind to GlmS, which catalyzes the first step in hexosamine metabolism by converting fructose-6P to glucosamine-6P using glutamine as a nitrogen source [77], yielding N-acetylglucosamine an essential building block of bacterial cell walls. Therefore, targeting this enzyme could lead to the inhibition of bacterial growth [78]. Predicted interactions between carnosic acid and GlmS are presented in Table S5.

#### 3.1.3. Pyruvate Kinase 2–Muscle Isoform

Cancer cells often rely on glycolysis to meet their high energy demands, whereas normal cells derive most of their energy from oxidative phosphorylation [79]. This difference in cell metabolism can be, therefore, exploited to target cancer cells. The muscle isoform of pyruvate kinase 2 (PKM2) is universally expressed in cancer cells and catalyzes the final step of glycolysis by transferring a phosphate group from phosphoenolpyruvate (PEP) to adenosine diphosphate (ADP), resulting in one molecule of pyruvate and one molecule of adenosine triphosphate (ATP). On the other hand, the remaining isozymes of pyruvate kinase are expressed in most normal tissues, so targeting PKM2 represents a viable way to selectively inhibit glucose metabolism in cancer cells [80]. Carnosic acid binds at the site where variations in two amino acid residues are present compared to PKM1, namely Ile389Met and Gln393Lys (Table S6). These variations result in a significant decrease in docking score as the best PKM1 isoform scores −65.1 A.U compared to −68.1 for the M2 isoform (Table 1), which may indicate that carnosic acid is indeed selective towards PKM2.

#### 3.1.4. Hemagglutinin HA1

**Figure 4:**
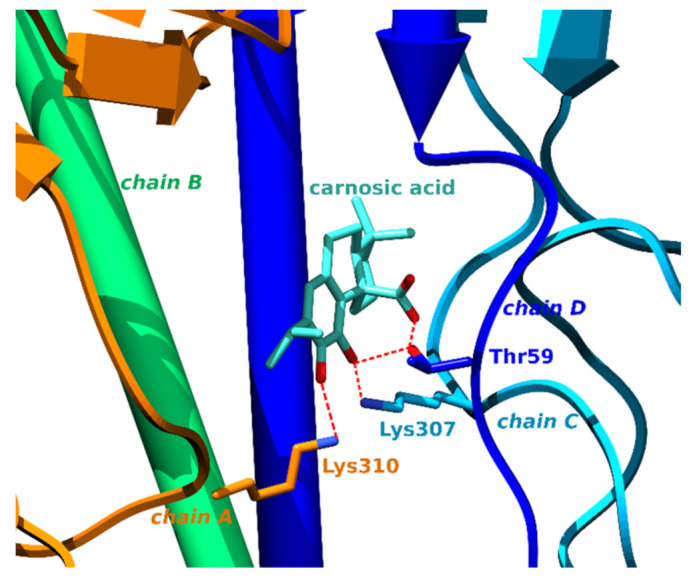
Carnosic acid glues together chains A, C and D of the HA glycoprotein. Carbons of carnosic acid are displayed as teal sticks, chain A in orange, chain B in green, chain C in sky blue and chain D in dark blue pipes and planks. Amino acids forming hydrogen bonds (denoted with red dotted lines) are displayed as sticks of matching colors.

Influenza virus hemagglutinin (HA) represents a surface glycoprotein that is critical for viral infectivity. It has multifunctional activity, allowing entry of the virus by binding to sialic acid at the surface of host cells, while also being responsible for the fusion of the viral envelope to the endosomal membrane [81]. Due to its importance, this protein forms a key target for neutralizing antibodies [82]. However, it is also possible to target it with small molecules such as arbidol [83]. Carnosic acid docks to a cavity in the HA trimer stem at the interface between the three protomers. This binding site is separate from the conserved epitope targeted by the neutralizing antibodies. The drug arbidol is known to stabilize the conformation of HA, thereby preventing the large conformational changes required for membrane fusion. This could potentially also be the case with carnosic acid, as it forms three hydrogen bonds, one with each protomer, and could thus act as a so-called molecular glue that binds the protomers together, making them nonfunctional (Figure 4, Table S7).

#### 3.1.5. HIV-1 and HIV-2 Protease

Human immunodeficiency viruses (HIV) protease is a retroviral aspartyl protease involved in the hydrolysis of several peptide bonds, which is essential for the life cycle and replication of HIV [84]. Small molecule inhibitors of HIV protease play a critical role in the effective treatment of acquired immunodeficiency syndrome AIDS, as they represent part of the highly active antiretroviral therapy (HAART). While HIV-1, carrier of the HIV-1 protease isoform, forms the most common subtype worldwide, HIV-2 remains mainly confined to West Africa and is also spreading in India [85,86]. However, the treatment of HIV-2 is more difficult than that of HIV-1, as most antiviral drugs have been developed for the HIV-1 isoform. HIV-2 proteases have also been found more resistant to small-molecule inhibition [87]. Moreover, dual infection with both isoforms is possible as well [88]. Consequently, novel inhibitors for both HIV proteases would be of great benefit. It has been shown that carnosic acid exhibits potent inhibition of the HIV-1 protease isoenzyme with an IC_90_ = 0.08 μg/mL [69]. Inhibition has not yet been experimentally demonstrated for the HIV-2 isoform; however, our studies suggest that carnosic acid is also capable of inhibiting this isoform. This finding can also be corroborated by the fact that the binding sites of both isoforms are very similar, with a sequence identity close to 70% and the ProBiS Z-score of 3.76 [58]. ProBiS Z-score measures the statistical and structural significance of local binding site similarity. Binding site alignments leading to ProBiS Z-Scores higher than 2 are considered to be very similar. In addition, all the equivalent binding site amino acid residues are of the same charge and polarity type. Overall, carnosic acid could prove to be a valuable starting point for the development of antivirals that would be effective against both strains of HIV.

**Figure 5:**
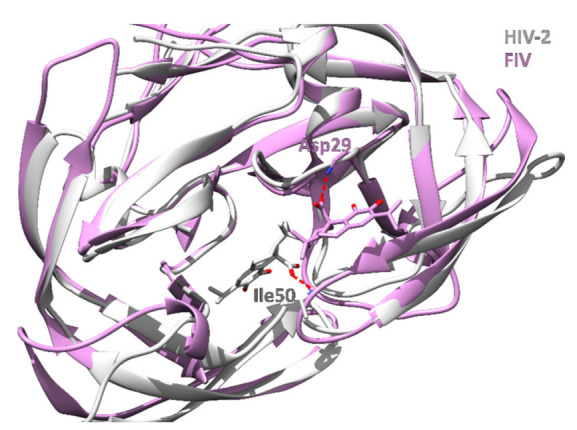
Comparison of HIV-2 (grey cartoon) and FIV (purple cartoon) protease. Carnosic acid in HIV-2 binds deep into the protease binding site and forms a hydrogen bond with the backbone nitrogen of Ile50 (red dotted line). On the other hand, carnosic acid is located closer to the protease surface in FIV and forms a hydrogen bond H-bond with the backbone nitrogen of Asp29 (red dotted line).

Interestingly, the inverse docking results also suggest a high binding ability of carnosic acid to the HIV-related feline immunodeficiency virus (FIV) protease, which causes an AIDS-like syndrome in cats. HIV-2 and FIV proteases possess a binding site similarity of 1.90, expressed by the ProBiS Z-score, and a general sequence similarity of 26%. The relatively different binding sites result in different binding positions of carnosic acid in HIV-2 and FIV proteases. In HIV-2, the ligand is positioned deeper in the major binding site, which is located between the two protomers (Figure 5, Tables S8, S13 and S15).

#### 3.1.6. Enhanced Intra-Cellular Survival Protein

Tuberculosis represents the leading cause of infectious death worldwide, primarily due to the emergence of multidrug-resistant tuberculosis and due to extensively drug-resistant strains of *Mycobacterium tuberculosis* [89]. Up-regulation of the enhanced intra-cellular survival (Eis) protein was found to be the sole cause of resistance to the aminoglycoside of last resort-kanamycin in approximately one-third of *Mycobacterium tuberculosis* isolates. Specifically, Eis represents an acetyltransferase responsible for *Mycobacterium tuberculosis* resistance to multiple aminoglycoside drugs. A distinctive property of Eis is that it acetylates the aminoglycoside drugs at multiple amine functional groups, preventing them from binding to their target, the ribosome. The simultaneous use of Eis inhibitors with anti-tuberculosis drugs may therefore provide a way to combat this resistance by restoring aminoglycoside drug activity [90]. Carnosic acid docks to the aminoglycoside binding pocket formed by the N-terminal domain to which also tobramycin binds, thereby suggesting the possibility of competitive inhibition of Eis by carnosic acid (Table S9) [91].

#### 3.1.7. Peroxisome Proliferator-Activated Receptor δ

The peroxisome proliferator-activated receptor (PPARδ) functions as a sensor for dietary and endogenous fats [92]. It regulates the transcription of genes associated with lipid and glucose metabolism. Specifically, it controls lipid degradation, transport, and storage, while also being associated with insulin secretion and resistance. PPARδ agonists have been shown beneficial in models of metabolic disorders in primates and may thus possess therapeutic potential in hyperlipidemia, atherosclerosis, obesity, and diabetes [93,94]. PPARδ is also associated with cancer by promoting chronic inflammation through increasing cyclooxygenase-2 (COX-2) expression and prostaglandin E2 production, leading to an increase in proinflammatory cytokine concentrations. Moreover, the ability of PPARδ to promote the use of fatty acids as the energy source may enhance cell survival and proliferation under harsh metabolic conditions often found in tumors. Therefore, PPARδ agonists may be useful in treating metabolic disorders, while antagonists may reduce inflammation-related disorders and slow down cancer progression.

**Figure 6:**
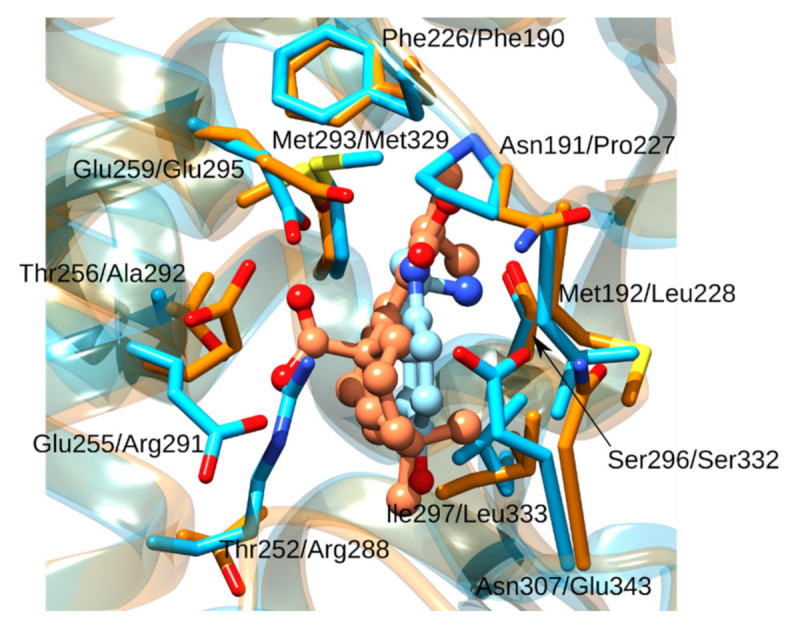
The Ω-pocket superimposition between PPARδ (orange cartoon and sticks) and PPARγ (blue cartoon and sticks). The first amino acid residue numbering corresponds to PPARδ, and the second to PPARγ. Serotonin is displayed using blue balls and sticks and the docked carnosic acid using orange balls-and-sticks. We emphasize the difference in amino acid residues Thr252 versus Arg288. Compared to Thr252 in PPAR_δ_, the large Arg288 in PPARγ would lead to stearic clashes with carnosic acid.

Whereas there are no experimental data that carnosic acid, carnosol, or rosmanol bind to PPARδ, it is known that both carnosol and carnosic acid represents agonists of the PPARγ isoform with half maximal effective concentration (EC_50_)values of 41 and 20 μM, respectively [95]. Carnosic acid docks to PPARδ in the same Ω-pocket where serotonin binds to PPARγ which also acts as agonists at PPARδ. The binding site possesses 62% amino acid sequence identity and a ProBiS Z-score of 3.36. From the superposition of PPARδ (with docked carnosic acid) and PPARγ (with serotonin) Ω-pockets, we observe that PPARγ produce steric clashes with carnosic acid (Figure 6, Table S10). However, due to experimental evidence, that carnosic acid indeed binds to PPARγ, we can predict that induced fitting effects play an important role. Because PPARδ possesses a smaller threonine in this place and because the overall binding site is similar, we can hypothesize that carnosic acid could bind even stronger to the PPARδ isotype as preliminary induced fitting would not be required.

#### 3.1.8. Glycogen Phosphorylase

**Figure 7:**
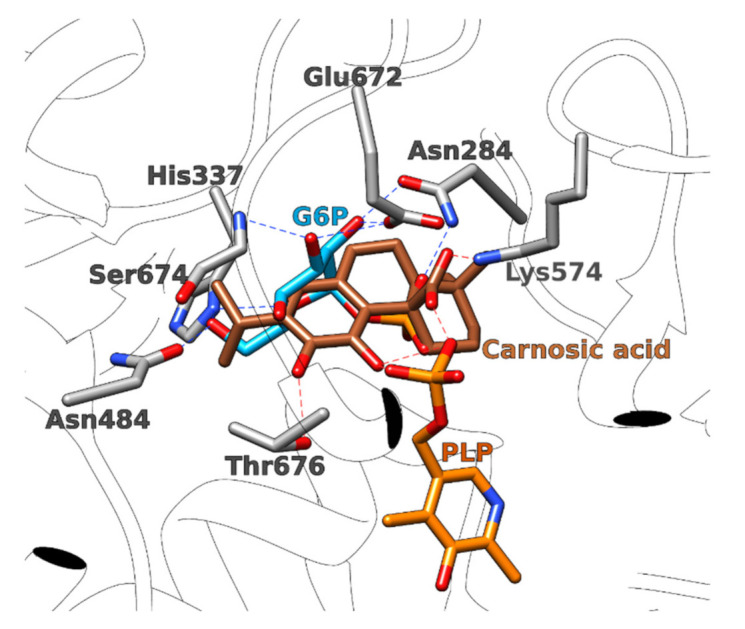
Glucose-6-phosphate (G6P, light blue sticks) overlapped with carnosic acid (brown sticks) in the catalytic binding site of glycogen phosphatase. Important amino acid residues are shown in grey sticks. Hydrogen bonds formed by carnosic acid are presented with red dotted lines, while the hydrogen bonds formed by glucose-6-phosphate are shown with blue dotted lines. The cofactor pyridoxal phosphate (PLP) is presented in orange sticks.

Glycogen phosphorylase (GP) is an enzyme that cleaves the non-reducing ends in the chain of glycogen to produce glucose-1-phosphate monomers which can be further converted to free glucose [96]. Because glycogen is an important source of blood glucose, GP represents a promising target for the treatment of type II diabetes, and its inhibitors have been shown effective in controlling blood glucose concentrations in animal studies [97]. GP can exist in two different forms that bind different regulatory molecules: the active phosphorylated (on Ser14) GPa and the non-phosphorylated GPb form. In addition, GP has been reported to bind compounds at four different binding sites, identified as: (a) the catalytic, (b) the allosteric (indole), (c) the novel allosteric, and (d) the inhibitory site (caffeine) [98]. In our study, carnosic acid docked to the catalytic site (a) of the GPb form, specifically to the α-D-glucose binding site, therefore, it might act as a competitive inhibitor with respect to glucose-1-phosphate (Figure 7) [96,99]. Glucose-1-phosphate forms hydrogen bonds with Glu672, Asn284, Ser674, His337, and Asn484, while the docked pose of carnosic acid binds to the cofactor pyridoxal phosphate with two hydrogen bonds, but also forming hydrogen bonds with Lys574 and Thr676 (Table S11).

#### 3.1.9. Tubulin

Tubulins represent protein monomers of microtubules, which form an essential component of the eukaryotic cytoskeleton [100,101]. They are involved in cell division as microtubules form mitotic spindles that are used by the cell to pull the chromosomes apart. Microtubules are produced by the polymerization of dimers of α- and β-tubulin that join together to form long hollow tubes called microtubules. Microtubule targeting agents such as chemotherapeutics vinblastine, colchicine, and paclitaxel bind to tubulin and disrupt microtubule dynamics, leading to a loss of function and to subsequent cell arrest or apoptosis. They can be classified into subgroups based on their binding site within the tubulin dimer: (a) the paclitaxel site at the β-tubulin in the microtubule lumen; (b) the vinblastine site at the interdimeric interface of two heterodimers; and (c) the colchicine site at the β-tubulin at the intra-subunit interface of a heterodimer. In our study, carnosic acid docked to the colchicine-binding site (c) (Table S12) and could therefore, like colchicine, potentially lead to microtubule depolymerization.

#### 3.1.10. Phosholipase A2

**Figure 8:**
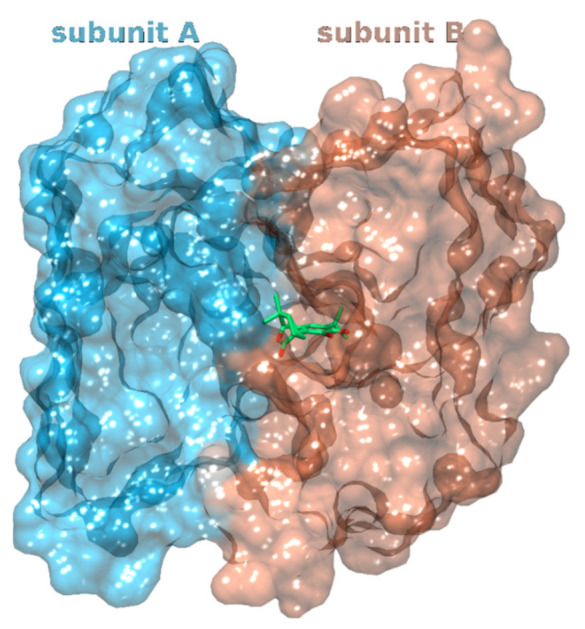
Carnosol (carbons denoted with green sticks) docks between subunits A (blue surface) and B (orange surface) of the phospholipase A2.

Phospholipases A2 (PLA2) represent enzymes that catalyze the hydrolysis of glycerophospholipids at the sn-2 position, releasing free fatty acids, including arachidonic acid. The action of PLA2 forms a crucial upstream step that increases free arachidonic acid levels and triggers the storm of eicosanoids, especially after inflammatory cell activation. Due to their involvement in the inflammatory response, PLA2 are thought to be associated with various diseases such as arthritis [102], cancer [103], coronary heart disease [104], and neurological disorders such as Alzheimer’s disease and multiple sclerosis [105]. In our study, carnosol docks between the two subunits of the dimer and forms a large hydrophobic and desolvated surface that is buried. Most of the carnosol molecule is located within the B subunit. (Figure 8, Table S14). Binding to identical active site as the alkyl portion of the tetrahedral mimic inhibitor MJ33.

#### 3.1.11. Vascular Endothelial Growth Factor Receptor 2

**Figure 9:**
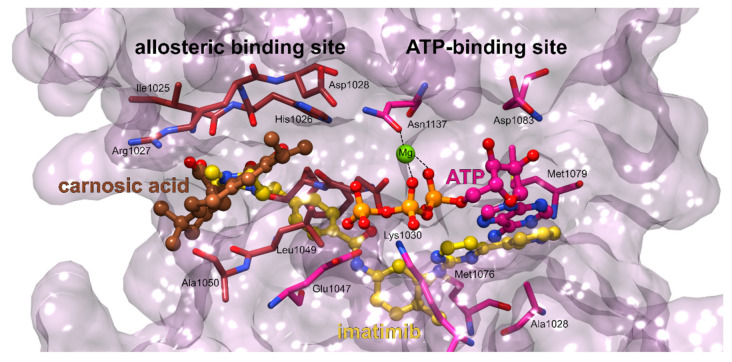
VEGFR2 binding sites. The VEGFR2 enzyme is presented with pink surface; the amino acid residues of the adenosine binding site are shown in pink sticks and of the lipophilic allosteric binding site in brown sticks. The docked carnosic acid located in the allosteric site displayed in brown balls and sticks and the ATP molecule in pink balls and sticks. The typical type II inhibitor imatimib, binding to both sites concurrently is depicted in yellow balls-and-sticks.

Vascular endothelial growth factor receptors (VEGFR) represent tyrosine kinase receptors for vascular endothelial growth factor (VEGF), a signaling protein critical in angiogenesis [106]. Because solid cancer tumors require an adequate blood supply to grow and metastasize, the inhibition of VEGFR signaling with small molecule drugs such as sorafenib or pazopanib is used as a well-established treatment in various cancers, since tumors cannot grow more than 2 mm without angiogenesis. VEGFR-2 plays an important role in cell migration and proliferation-two crucial steps of angiogenesis. Carnosic acid docks to the binding site representative of type II kinase inhibitors. In general, type II inhibitors, such as sorafenib and lenvatinib, are often more specific than those targeting only the ATP binding site [107,108]. They represent a class of compounds that capture kinases in an inactive form and occupy both the adenine region (of ATP) as well as a hydrophobic pocket adjacent to the ATP binding site [109]. However, due to the small size of carnosic acid, only the hydrophobic binding site is actually occupied (Figure 9, Table S16). This is consistent with the experimental finding that carnosic acid or carnosol actually do not possess a measurable inhibitory activity against VEGFR2 [109]. However, given the strong interaction measured between carnosic acid and VEGFR2 applied in the inverse docking method, carnosic acid could potentially serve as a base compound to which a specific ring system region would be added to also target the adenine binding site.

#### 3.1.12. Aspartate Carbamoyltransferase

*Plasmodium falciparum* and *Trypanosoma cruzi* represent parasites that cause malaria and Chagas disease, respectively [110]. Aspartate carbamoyltransferase is an enzyme involved in pyrimidine biosynthesis that catalyzes the formation of phosphate and N-carbamoyl L-aspartate from carbamoyl phosphate and L-aspartate. Reproduction of both parasites requires a sufficient supply of purines, as they form the building blocks of nucleic acid molecules. Recent studies in *Plasmodium falciparum* have shown that aspartate carbamoyltransferase represents a suitable drug target, as its inhibition leads to a reduction in parasite growth [111]. Carnosic acid docks in the aspartate carbamoyltransferase of both *Plasmodium falciparum* and *Trypanosoma cruzi* at the interface between the protomers in the carbamoyl phosphate domain, where the carbamoyl phosphate substrate binds (Tables S17 and S18) [112].

### 3.2. Inverse Docking of Rosmarinic Acid

Table 2 lists the top-scoring protein–ligand complexes based on the cut-off criterion of 3.5 σ. We focus only on protein targets related to human health, i.e., we present only proteins from humans and mammals, as well as proteins from pathogenic microorganisms. As before, where data were available, we redocked ligands/drugs known to bind to the presented protein targets using a procedure analogous to inverse docking. These results, presented in Table S1, show that the docking scores of known ligand/drugs are in all cases worse than those of rosemarinic acid. Again, this may indicate an already strong binding affinity of rosmarinic acid, although it has not yet been rationally optimized for these specific protein targets.

#### 3.2.1. Coagulation Factor X

**Figure 10:**
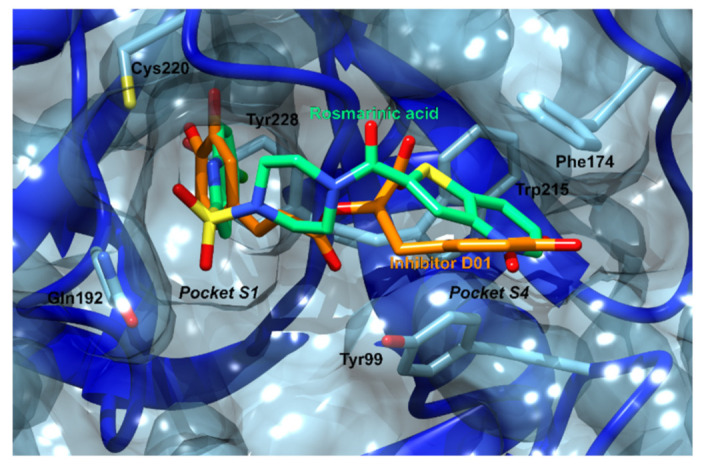
Factor Xa binding site. Factor Xa is shown in blue ribbons and surface, its important amino acid residues in blue sticks. Rosmarinic acid (green carbons) is docked in the same binding site as the one occupied by a known inhibitor (orange sticks) with a PDB ID: D01. The caffeic acid part of rosmarinic acid docks to the S1 pocket, and the 3,4-dihydroxyphenyllactic acid moiety to the S4 pocket.

Factor X represents an enzyme involved in the coagulation cascade that, when activated by the hydrolysis of factor Xa, claves prothrombin to the active thrombin, which in turn converts soluble fibrinogen to insoluble fibrin strands [115]. The role of factor X is particularly important because it is the first enzyme where the intrinsic and extrinsic coagulation pathways converge. Drug manipulation of the coagulation cascade is extremely important in modern medicine, since reducing excessive coagulation is critical for preventing diseases such as myocardial infarction and ischemic stroke, which belong among the leading causes of death and disability in the Western world [116,117]. Oral inhibitors of factor X, such as rivaroxaban, are already successfully used in clinical practice [118]. Rosmarinic acid docks in an analogous manner to a number of sulfonamide factor X inhibitors (Table S19) [119]. Its caffeic acid ring binds to the S1 pocket, while its 3,4-dihydroxyphenyllactic acid moiety binds to the S4 pocket (Figure 10).

#### 3.2.2. Phospholipase A2

Similar to the case of carnosol, our inverse docking algorithm also detected a strong binding to the enzyme phospholipase A2. This is consistent with existing experimental evidence, as the PDB contains a snake toxin phospholipase A2 homolog (PDB ID: 3QNL) bound with rosmarinic acid, and this complex was also applied later on in our study to validate the inverse docking algorithm by redocking. Compared to the main active site, its binding site is located in a different region between the dimer site where the MJ33 inhibitor was reported to bind and where carnosol was docked in this study (Table S20). We have here an interesting case where two rosemary compounds potentially inhibit the same enzyme.

#### 3.2.3. Matrix Metalloproteinase-3

**Figure 11:**
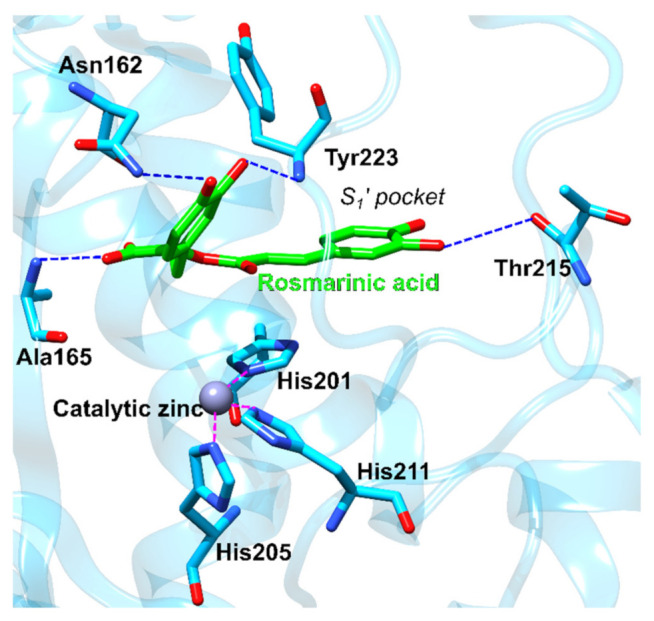
Rosmarinic acid docked in MMP3 (blue ribbons). The rosmarinic acid docks near the catalytic zinc ion and one of the catechol groups positions inside the S_1′_ selectivity pocket. Important amino acid residues are shown in blue sticks, hydrogen bonds are denoted with dotted blue lines and coordinative bonds with dotted purple lines.

Matrix metalloproteinase-3 (MMP-3) represents a zinc-dependent endopeptidase Matrix metalloproteinase-3 (MMP-3) represents a zinc-dependent endopeptidase involved in extracellular matrix remodeling [120]. It is, therefore, required for physiological processes such as embryonic development and reproduction and is also involved in various pathological processes. Moreover, MMP-3 can also activate other metalloproteinases, enter cell nuclei, and control gene expression. Excessive activation of MMPs can lead to excessive degradation of the extracellular matrix and to numerous pathological conditions such as arthritis, multiple sclerosis, aneurysms, as well as the spread of metastatic cancer. Furthermore, it has been shown that following a traumatic brain injury, MMP-3 levels can also increase and cause additional damage to the blood–brain barrier [70]. The discovery of novel small molecule inhibitors of MMP-3 is, therefore, of great importance for the treatment of numerous diseases [120]. The 3,4-dihydroxyphenyllactic moiety of rosmarinic acid docks to the catalytic region, but it is too far from the catalytic zinc ion to form direct interactions with it (Figure 11, Table S21). The caffeic acid moiety docks to the S1’ pocket that delimits the active site. The S1’ pocket is known to confer the selectivity of compounds towards different matrix metalloproteinases. Therefore, compounds that interact within the S1’ pocket and not with the catalytic zinc could selectively inhibit one particular MMP without affecting the activities of the remaining ones.

#### 3.2.4. Farnesyl Pyrophosphate Synthase

**Figure 12:**
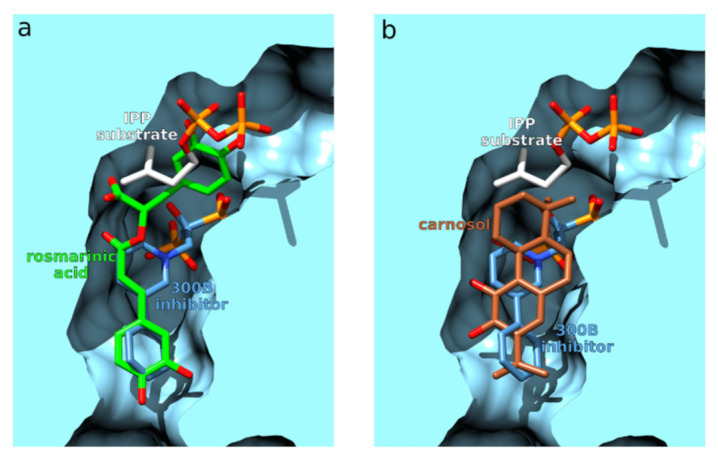
Comparison of ligand binding to farnesyl pyrophosphate synthase (blue surfaces). (**a**) Comparison between the crystal ligand 300B (blue carbons) and rosmarinic acid (green carbons) binding. (**b**) Comparison between the crystal ligand 300B (blue carbons) and carnosol (brown carbons) binding. The enzyme substrate isopentenyl pyrophosphate (IPP) was not present during the inverse docking but is shown for comparison purposes using white carbons.

*Leishmania major* represents an intracellular, pathogenic, parasitic organism that causes cutaneous leishmaniasis. The World Health Organization stated that leishmaniasis is one of the most neglected diseases. Moreover, 350 million people are considered at risk of contracting the disease, approximately 12 million people are infected worldwide, and an estimated two million new cases occur each year [121]. Farnesyl pyrophosphate synthase (FPPS) represents an important enzyme involved in the biosynthesis of ergosterol in Leishmania parasites. Antiparasitic compounds targeting the ergosterol biosynthesis play an important role in the treatment of leishmaniasis, and the inhibition of FPPS has been shown largely effective against the related *Leishmania donovani* [122]. Interestingly, a study [114] showed that carnosic acid and carnosol form potent inhibitors of human FPPS, with IC_50_ values of 20.0 and 13.3 μM, respectively. It also demonstrated that inhibition of the human form of the enzyme leads to the induction of apoptosis in pancreatic cell lines by downregulating RAS prenylation. *Leishmania major* FPPS is not among the 0.05% best scoring proteins of rosemary diterpenes and is not listed in Table 1. However, it still scored extremely high with carnosol (−60.4), which is within the 3.0σ. Thus, as with phospholipase A2, we have yet another interesting case of two rosemary polyphenols potentially inhibiting the same enzyme. Both rosmarinic acid and carnosol bind approximately to the same protein space, with portions of the ligands occupying the same region as the reported *Leismania minor* FPPS inhibitor 1-(2-hydroxy-2,2-diphosphonoethyl)-3-phenylpyridinium (300B) (Figure 12, Table S22). Part of the rosmarinic acid enters the substrate-binding region where the substrate isopentenyl pyrophosphate is present in an uninhibited enzyme.

#### 3.2.5. Glutamate Dehydrogenase 1 and Glutaminase

Glutaminase and glutamate dehydrogenase 1 (GDH1) represent enzymes that are both part of the glutaminolysis pathway. Glutaminolysis begins with the conversion of glutamine to glutamate by glutaminase, while the next step is catalyzed by GDH, which converts glutamate to 2-oxoglutarate. The two enzymes play a crucial role in nitrogen and carbon metabolism, as the product 2-oxoglutarate feeds the citric acid cycle. Numerous cancer cells rely on increased glutaminolysis to meet their energy requirements. It has thus been shown that the inhibition of glutaminase and GDH1 by small molecules leads to a decreased viability of cancer cells in vivo and in vitro. Consequently, they form promising targets for cancer treatment [123,124]. It has been already shown that the plant compounds from green tea epigallocatechin gallate and epicatechin gallate strongly inhibit GDH [125,126,127]. According to our inverse docking procedure, rosmarinic acid is located at a different binding site than the green tea compounds. It binds at hexameric 2-fold axes between the dimers of the GDH subunits, where known inhibitors such as bithionol are also located (Table S23) [127].

**Figure 13:**
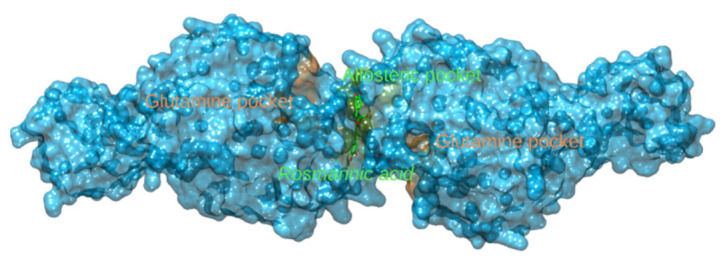
Glutaminase binding sites. Two protomers forming glutaminase are shown on blue surfaces. The main glutamine substrate-binding pockets are highlighted in orange surfaces, whereas rosmarinic acid (carbons denoted with green sticks) docks into the allosteric binding site (green surfaces) formed between the two glutamase protomers.

Rosmarinic acid binds to the allosteric pocket formed at the interface between the two dimers of glutaminase (Figure 13). In numerous crystal structures of glutaminase in the PDB, co-crystallized inhibitors have occupied this binding site, e.g., 3UO9, 3VOZ, and 3VP1 (Table S24) [128,129].

### 3.3. Method Validation

#### 3.3.1. Redocking Procedure

To validate the inverse molecular docking procedure, a redocking study was performed using all available protein complexes from the PDB containing rosmarinic acid (PDB structures: 6MQD, 3QNL, and 4PWI). An analogous redocking procedure using the investigated diterpene structures could not be performed because protein structures containing carnosic acid, carnosol, or rosmanol do not yet exist in the PDB. Redocking of rosmarinic acid was performed by first removing the ligand from the binding site. Then, the CANDOCK algorithm was used with identical settings for inverse molecular docking to bind rosmarinic acid to the binding site defined by the crystal structure. The actual binding site definition was again identical to the one found in the ProBiS-Dock Database. To evaluate the success of the redocking procedure, the root-mean-square deviation (RMSD) of all heavy atoms between the co-crystallized and the redocked rosmarinic acid was measured.

**Figure 14:**
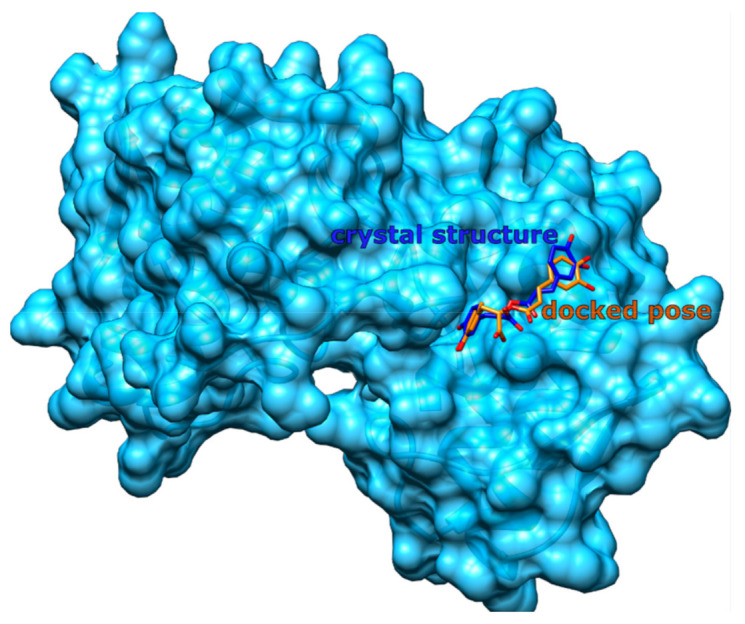
A successful redocking of rosmarinic acid to the crystal structure of phospholipase A2 (PDB ID: 3QNL) from the snake venom (depicted in blue ribbons and transparent surfaces). The stick structure of rosmarinic acid with blue carbons represents the native ligand position found in the crystal structure, while the structure with orange carbons displays the redocked structure. Hydrogen atoms are not shown for clarity. The RMSD between the two rosmarinic acid structures is 1.3 Å.

From a molecular docking perspective, rosmarinic acid represents a problematic molecule, because it contains a high number, namely seven, rotatable bonds, which makes it difficult for the docking algorithms to consistently identify the correct conformer of this molecule. This problem is reflected in the fact that we obtained a low RMSD value of 1.3 Å only with the PDB structure 3QNL, which is a snake venom-derived phospholipase A2 structure [113], compared to the original crystal structure (Figure 14). The redocking procedure was not successful for 4PWI or 6MQD structures with significantly larger RMSD values (not shown), implying that the correct pose was not detected with the CANDOCK docking algorithm. However, based on a successful redock with 3QNL and on the fact that the docking algorithm identified numerous targets that have already been also experimentally confirmed for both rosemary diterpenes as well as rosmarinic acid, we are confident that the method is capable of recognizing correct protein targets to large extent.

#### 3.3.2. Validation Using ROC, EF, and PC

**Figure 15:**
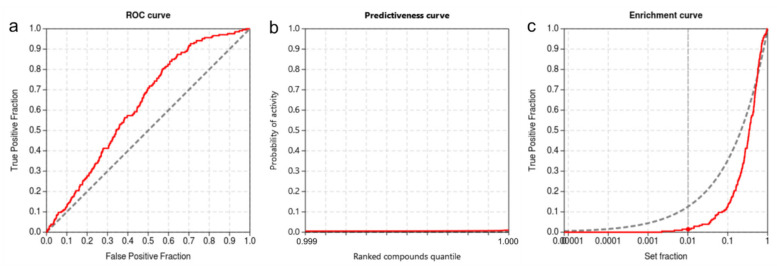
Validation of the inverse molecular docking protocol using rosmarinic acid: (**a**) the ROC curve, with the ROC AUC of 0.627; (**b**) predictiveness curve, from which the TG of 0.171 is determined; (**c**) enrichment curve.

We performed the inverse molecular docking using the CANDOCK algorithm on all proteins from the ProBiS-Dock database, including 206 experimentally confirmed targets of rosmarinic acid, whose measured IC_50_ values were < 10 μM [62]. The ability of our protocol to distinguish the confirmed protein targets from proteins that reportedly do not bind rosmarinic acid was evaluated using the metrics established in the virtual screening community (Figure 15). It was successful in discriminating between the true and false targets of rosmarinic acid (ROC AUC of 0.627). The early detection of protein targets was assessed by the BEDROC of 0.071, by the RIE of 1.403, and by the EF 1% of 1.46, which is satisfactory. The inverse molecular docking protocol based on the CANDOCK algorithm resulted in score variations for the detection of true target proteins (TG determined from PC has a value of 0.171), which in combination with ROC AUC above 0.6 indicates that the protocol provides good results in agreement with the experiments [61]. Moreover, our inverse molecular docking protocol has been already extensively validated by Fine and Konc et al. [55], Furlan et al. [50,130], Kores et al. [49,131], and Jukič et al. [132] using different molecules of interest.

### 3.4. In Silico Prediction of Pharmacokinetic Properties

In concurrence with experimental findings [31], the SwissADME web server [54] indeed predicts that carnosic acid, carnosol, and rosmanol all exhibit high gastrointestinal absorption (data shown in Supplementary Materials). On the contrary, the server predicts low gastrointestinal absorption for rosmarinic acid, which is again in line with the available in vivo data [43]. All compounds are predicted to be moderately soluble. Diterpenes are overall predicted as quite lipophilic, with a consensus score of logP above 3.5 for carnosic acid and carnosol, and 2.9 for rosmanol. Rosmarinic acid, as expected, due to the large number of polar functional groups, exhibits a much lower logP value of 1.2. Interestingly, carnosol is the only molecule predicted to penetrate the blood–brain barrier, however experimental studies on rat animal models show that carnosic acid also effectively penetrates the blood–brain barrier [32]. The SwissADME output is presented in its entirety in the Supplementary Materials.

## 4. Discussion

Natural plant-based compounds play an important role in the development of novel drugs as they may possess several advantages over conventional synthetic compounds, namely, fewer side effects, lower long-term toxicity, and versatile biological effects [130]. We report the potential targets of the major compounds from *Rosmarinus officinalis*, including the diterpenes carnosic acid, carnosol, and rosmanol, as well as the polyphenolic ester rosmarinic acid. Their targets were identified in silico using an inverse molecular docking approach. All four compounds were individually docked to all non-redundant holo-proteins available in the PDB. To identify the binding sites of each protein in advance, we applied the recently developed ProBiS-Dock Database—a freely available repository of binding sites between small ligands and proteins. Thereby, the docking procedure was limited to binding sites already known to bind at least one drug-like small-molecule ligand or to binding sites exhibiting a high similarity with the already known binding sites. Moreover, we used the novel CANDOCK algorithm, which employs a fragment-based docking approach with maximum clique and a knowledge-based scoring function.

Due to the similar molecular structure and docking/scoring values, we combined the results of all three investigated diterpenes into a single set (Table 1). We identified numerous human/mammalian proteins that could explain the observed anticarcinogenic activities of rosemary diterpenes. The best docking score was obtained for the complex between carnosic acid and the proto-oncogene K-Ras G12C. Moreover, the anticarcinogenic activities can also be explained by the potential binding of rosemary diterpenes to pyruvate kinase, PPARδ, tubulin, VEGFR2, and phospholipase A2. In general, phospholipase A2 has also been strongly implicated in inflammation-related disorders, so its inhibition may be likewise beneficial in arthritis, coronary artery disease, or dementia. Due to the identification of potential binding of the investigated diterpenes to glycogen phosphorylase, which facilitates glucose production, these compounds may be also useful in the treatment of type II diabetes. Furthermore, rosemary diterpenes exhibit antiviral activities.

From previous experimental studies, it is known that carnosol strongly inhibits HIV-1 protease. However, we also found out that rosemary diterpenes may bind strongly to the HIV-2 enzyme isotype. These compounds therefore likely represent a good starting point for the development of drugs against AIDS that could treat concurrent infections with HIV-1 and HIV-2. Interestingly, all diterpenes also yield good docking scores when bound to the feline immunodeficiency virus (FIV) protease, which is strongly related to HIV proteases, suggesting their potential utility in veterinary medicine. Finally, we have also found out that these compounds can bind to HA1 of the influenza A virus, potentially reducing its infectivity.

The antibacterial activity of investigated diterpenes can be explained by our discovery that they can bind to the enzyme glucosamine-fructose-6-phosphate aminotransferase in *Escherichia coli*, which is critical for the first step of hexosamine metabolism responsible for bacterial growth. Encouragingly, we have also found out that they can bind to the Eis protein of *Mycobacterium tuberculosis*, which confers resistance to aminoglycoside drugs, rendering them inactive. Therefore, the inhibition of this enzyme in conjunction with tuberculosis treatment could be beneficial in reducing the bacterial resistance to these drugs.

The investigated diterpenes also displayed binding to aspartate carbamoyltransferase of two different pathogenic parasites-*P. falciparum* and *T. cruzi*. *P. falciparum* represents the causative agent of malaria, while *T. cruzi* causes Chagas disease. Inhibition of this enzyme results in the inability of the two parasites to produce pyrimidines, limiting their biosynthesis of new nucleic acids.

Like diterpenes, rosmarinic acid also shows binding to proteins involved in carcinogenesis, namely matrix metalloproteinase-3 and phospholipase A2. Interestingly, all four compounds display very favorable binding scores for the enzyme phospholipase A2, which could provide a possible explanation for the strong anti-inflammatory effects of rosemary. According to our results, rosmarinic acid may also interfere with the glutaminolysis pathway, as it forms top-scoring complexes with two related enzymes—glutaminase and glutamate dehydrogenase. Inhibition of this pathway by small-molecule drugs has been indeed shown to reduce cancer cell viability. Moreover, the complex between rosmarinic acid and coagulation factor X yielded the best scoring result. Regulating blood clotting with drugs is of utmost importance, as reducing excessive blood clotting is crucial in preventing diseases such as heart attacks and ischemic strokes, which belong among the leading causes of death and disability in the Western world. Furthermore, rosmarinic acid might also possess antiparasitic activity as its binding to farnesyl pyrophosphate synthase (FPPS) of *Leishmania major* obtained a favorable docking score. This parasite causes zoonotic cutaneous leishmaniasis, and inhibition of the FPPS prevents the biosynthesis of ergosterol.

The results of this study will facilitate future molecular dynamics studies. Therein, we plan to investigate the dynamic binding patterns of prior parameterized rosemary compounds to the notable protein targets identified here. The molecular dynamics observations will be extended with the linear interaction energy as well as linear response approximation calculations to obtain the binding free energy values, which will then be compared with drug ligands already known to bind to the protein targets described here.

## 5. Conclusions

Using an in silico inverse molecular docking procedure, we identified protein targets that could explain the observed pharmacological activities of rosemary or its major polyphenolic constituents. By identifying protein structures to which carnosic acid, carnosol, rosmanol, and rosmarinic acid can bind, we provide possible explanations for the observed anticarcinogenic, anti-inflammatory, antidiabetic, antiviral, and antibacterial activities of rosemary. In addition, using this methodology we were able to predict new effects of these compounds that have not yet been reported, namely their anticoagulant and antiparasitic activities. Lastly, we believe that our research can form the basis for the development of novel drugs, where the rosemary compounds studied here could serve as a starting point for efficient drug design.

## Figures and Tables

**Table 1:** Best scoring human, mammalian, and pathogen protein targets of rosemary diterpenes carnosic acid, carnosol, and rosmanol. Docking scores independent of the organism or type of protein are collected in Supplementary Materials in Table S2.

Rank	PDB ID with Chain	Ligand	Predicted Ligand Docking Score (arb. Units)	Protein Name	Organism	Protein Function and Disease Correlation	Reported Experimental Correlation of Protein and Ligand
1	4lucB	Carnosic acid	−69.9	K-Ras G12C	*Homo sapiens*	Controls cell proliferation and differentiation. Its gene is a proto-oncogene.	[65]
2	3oojA	Carnosic acid	−68.2	Glucosamine- fructose-6-phosphate aminotransferase	*Escherichia coli*	Catalyzes the first step in hexosamine metabolism and is needed for *E. coli* growth and infection spread.	[66,67,68]
3	3srdD	Carnosic acid	−68.1	Pyruvate kinase M2	*Homo sapiens*	Catalyzes the last step in the glycolysis. Important in providing ATP to cancer cells.	
4	1kenA	Carnosic acid	−66.9	Hemagglutinin HA1	*Influenza A virus*	Enables viral entry into cells causing the flu.	
5	2hpeA	Carnosic acid	−65.0	HIV-2 protease	*Human immunodeficiency virus 2*	Hydrolyzes peptide bonds leading to functional proteins essential for HIV infectivity.	[69]
6	4jd6C	Carnosic acid	−64.8	Enhanced intracellular survival protein	*Mycobacterium tuberculosis*	Acetylates amine groups in aminoglycoside drugs, thus preventing the binding to the ribosome, leading to *M. tuberculosis* resistance.	
7	5u46A	Carnosic acid	−64.7	Peroxisome proliferator activated receptor delta	*Homo sapiens*	Regulates lipid catabolism and its transport and storage and is also associated with insulin secretion and resistance. It is implicated in metabolic disorders and cancer.	γ isoform [70]
8	3mt7A	Carnosic acid	−64.5	Glycogen phosphorylase	*Oryctolagus cuniculus*	Breaks the non-reducing ends in the chain of glycogen that enables glucose production. Its inhibition can manage type II diabetes.	
9	3rycC	Carnosic acid	−64.2	Tubulin	*Rattus norvegicus*	Involved in cell division as it forms microtubules which in turn form mitotic spindles that pull chromosomes apart during cell division. Tubulin targeting is used in cancer treatment.	
10	2j9kB	Carnosic acid	−63.5	HIV-1 protease	*Human immunodeficiency virus 1*	Hydrolyzes peptide bonds leading to functional proteins essential to HIV infectivity.	[69]
11	1fxfB	Carnosol	−63.3	Phospholipase A2	*Sus scrofa*	Catalyzes the hydrolysis of glycerophospholipids thus releasing free fatty acids, including arachidonic acid. Its action is implicated in several inflammation-based diseases such as arthritis, coronary artery disease, Alzheimer’s and cancer.	
12	3ogpA	Carnosic acid	−63.3	FIV Protease	*Feline immunodeficiency virus*	Hydrolyzes peptide bonds leading to functional proteins essential to FIV infectivity in cats.	
13	2p2hA	Carnosic acid	−63.1	Vascular endothelial growth factor receptor 2	*Homo sapiens*	Signal protein crucial in angiogenesis. Its inhibition is used in cancer treatment.	Negative: [71]
14	5ilqC	Carnosic acid	−63.0	Aspartate carbamoyltransferase	*Plasmodium falciparum*	Enzyme involved in pyrimidine biosynthesis, crucial for *Plasmodium falciparum* (causative agent of malaria) survival and replication.	
15	4iv5A	Carnosic acid	−62.8	Aspartate carbamoyltransferase	*Trypanosoma cruzi*	Enzyme involved in pyrimidine biosynthesis, crucial for *Trypanosoma cruzi* (causative agent of Chagas disease) survival and replication	

**Table 2:** Best scoring mammalian, human, and pathogen protein targets of rosmarinic acid. Docking scores independent of the organism or type of protein are collected in Supplementary Materials in Table S3.

Rank	PDB ID with Chain	Predicted Ligand Docking Score (arb. Units)	Protein Name	Organism	Protein Function and Disease Correlation	Reported Experimental Correlation of Protein and Ligand
1	2d1jA	−86.1	Coagulation factor X	*Homo sapiens*	Serine endopeptidase is involved in the coagulation cascade. Its deficiency leads to a bleeding disorder. Its inhibitors are popular anticoagulants.	
2	1fxfB	−84.8	Phospholipase A2	*Sus scrofa*	Catalyzes the hydrolysis of glycerophospholipids thus releasing free fatty acids, including arachidonic acid. Its action is implicated in several inflammation-based disease such as arthritis, coronary artery disease, Alzheimer’s and cancer.	[113]
3	2jt5A	−84.5	Matrix metalloproteinase-3	*Homo sapiens*	Zinc-dependent endopeptidase which is involved in the remodeling of the extracellular matrix. Involved in arthritis, multiple sclerosis, aneurysms, and the spread of metastatic cancer. After traumatic brain injury, matrix metalloproteinase-3 (MMP-3) concentrations increase and lead to additional damage to the blood–brain barrier.	
4	4jzbA	−83.2	Farnesyl pyrophosphate synthase	*Leishmania major*	Farnesyl pyrophosphate synthase (FPPS) is an essential enzyme involved in the biosynthesis of ergosterol in leishmania parasites, the causative agents of leishmaniasis.	[114]
5	3qmuB	−80.2	Glutamate dehydrogenase 1	*Bos Taurus*	Part of the glutaminolysis pathway, playing a crucial role in nitrogen and carbon metabolism. Inhibition leads to in vivo and in vitro reduced viability of cancer cells.	
6	5fi6A	−77.6	Glutaminase	*Homo sapiens*		

## Data Availability

All data generated or analyzed during this study are included in the published article.

## Supplementary Materials

The following are available online at https://www.mdpi.com/article/10.3390/foods11010067/s1. Table S1: Best docking scores of ligands/drugs known to bind to a specific target. Table S2: Best docking scores for carnosol, carnosic acid and rosmanol. Table S3: Best docking scores for rosmarinic acid. Table S4–S24: Interaction of compounds with respective targets presented in Table 1; Table 2. The SwissADME output file can be found in the file swissadme_output.xlsx supplied as part of the Supplementary Materials.

## Abbreviations

SMILES	Simplified Molecular-Input Line-Entry System
CANDOCK	Chemical Atomic Network based Docking
CHARMM	Chemistry at Harvard Macromolecular Mechanics
ROC	Receiver operating characteristics curve
PC	Predictiveness curve
TPF	True positive fraction
FPF	False positive fraction
PDB	Protein Data Bank
ROC AUC	Area under the receiver operating characteristics curve
EF	Enrichment factor
BEDROC	Boltzmann-enhanced discrimination of ROC
RIE	Robust initial enhancement
TG	Total gain
Eis	Enhanced intracellular survival
HIV	Human immunodeficiency virus
FIV	Feline immunodeficiency virus
RAS/MAPK	Rat sarcoma/mitogen-activated protein kinase
GTP	Guanosine triphosphate
GlmS	Glucosamine/fructose-6-phosphate aminotransferase
PKM	Pyruvate kinase M
PEP	Phosphoenolpyruvate
ADP	Adenosine diphosphate
ATP	Adenosine triphosphate
HA	Hemagglutinin
AIDS	Acquired immunodeficiency syndrome
HAART	Highly active antiretroviral therapy
PPAR	Peroxisome proliferator-activated receptor
COX-2	Cyclooxygenase-2
EC_50_	Half maximal effective concentration
GP	Glycogen phosphorylase
PLA2	Phospholipase A2
VEGFR	Vascular endothelial growth factor receptor
VEGF	Vascular endothelial growth factor
MMP	Matrix metalloproteinase
FPPS	Farnesyl pyrophosphate synthase
IPP	Isopentenyl pyrophosphate
GDH1	Glutamate dehydrogenase 1
RMSD	Root-mean-square deviation

## References

[1] Begum A., Sandhya S., Ali S.S., Vinod K.R., Reddy S., Banji D. (2013). An in-depth review on the medicinal flora *Rosmarinus officinalis* (Lamiaceae). Acta Sci. Pol. Technol. Aliment..

[2] Al-Sereiti M.R., Abu-Amer K.M., Sena P. (1999). Pharmacology of rosemary (*Rosmarinus officinalis* Linn.) and its therapeutic potentials. IJEB.

[3] Perez-Fons L., Garzon M.T., Micol V. (2009). Relationship between the antioxidant capacity and effect of rosemary (*Rosmarinus officinalis* L.) polyphenols on membrane phospholipid order. J. Agric. Food Chem..

[4] Yu M.-H., Choi J.-H., Chae I.-G., Im H.-G., Yang S.-A., More K., Lee I.-S., Lee J. (2013). Suppression of LPS-induced inflammatory activities by *Rosmarinus officinalis* L.. Food Chem..

[5] Bakırel T., Bakırel U., Keleş O.Ü., Ülgen S.G., Yardibi H. (2008). In vivo assessment of antidiabetic and antioxidant activities of rosemary (*Rosmarinus officinalis*) in alloxan-diabetic rabbits. J. Ethnopharmacol..

[6] Bozin B., Mimica-Dukic N., Samojlik I., Jovin E. (2007). Antimicrobial and antioxidant properties of rosemary and sage (*Rosmarinus officinalis* L. and *Salvia officinalis* L., Lamiaceae) essential oils. J. Agric. Food Chem..

[7] Tai J., Cheung S., Wu M., Hasman D. (2012). Antiproliferation effect of Rosemary (*Rosmarinus officinalis*) on human ovarian cancer cells in vitro. Phytomedicine.

[8] Valdés A., García-Cañas V., Rocamora-Reverte L., Gómez-Martínez Á., Ferragut J.A., Cifuentes A. (2013). Effect of rosemary polyphenols on human colon cancer cells: Transcriptomic profiling and functional enrichment analysis. Genes Nutr..

[9] Yesil-Celiktas O., Sevimli C., Bedir E., Vardar-Sukan F. (2010). Inhibitory effects of rosemary extracts, carnosic acid and rosmarinic acid on the growth of various human cancer cell lines. Plant Foods Hum. Nutr..

[10] Singletary K., MacDonald C., Wallig M. (1996). Inhibition by rosemary and carnosol of 7,12-dimethylbenz[a]anthracene (DMBA)-induced rat mammary tumorigenesis and in vivo DMBA-DNA adduct formation. Cancer Lett..

[11] Huang M.-T., Ho C.-T., Wang Z.Y., Ferraro T., Lou Y.-R., Stauber K., Ma W., Georgiadis C., Laskin J.D., Conney A.H. (1994). Inhibition of Skin Tumorigenesis by Rosemary and Its Constituents Carnosol and Ursolic Acid. Cancer Res..

[12] Lešnik S., Furlan V., Bren U. (2021). Rosemary (*Rosmarinus officinalis* L.): Extraction techniques, analytical methods and health-promoting biological effects. Phytochem. Rev..

[13] Okamura N., Fujimoto Y., Kuwabara S., Yagi A. (1994). High-performance liquid chromatographic determination of carnosic acid and carnosol in *Rosmarinus officinalis* and *Salvia officinalis*. J. Chromatogr. A.

[14] Johnson J.J. (2011). Carnosol: A promising anti-cancer and anti-inflammatory agent. Cancer Lett..

[15] Masuda T., Inaba Y., Maekawa T., Takeda Y., Tamura H., Yamaguchi H. (2002). Recovery Mechanism of the Antioxidant Activity from Carnosic Acid Quinone, an Oxidized Sage and Rosemary Antioxidant. J. Agric. Food Chem..

[16] Collins M.A., Charles H.P. (1987). Antimicrobial activity of Carnosol and Ursolic acid: Two anti-oxidant constituents of *Rosmarinus officinalis* L.. Food Microbiol..

[17] Shin H.-B., Choi M.-S., Ryu B., Lee N.-R., Kim H.-I., Choi H.-E., Chang J., Lee K.-T., Jang D.S., Inn K.-S. (2013). Antiviral activity of carnosic acid against respiratory syncytial virus. Virol. J..

[18] Pukl M., Umek A., Pariš A., Štrukelf B., Renko M., Korant B.D., Turk V. (1992). Inhibitory effect of carnosolic acid on HIV-1 protease. Planta Med..

[19] Bai N., He K., Roller M., Lai C.-S., Shao X., Pan M.-H., Ho C.-T. (2010). Flavonoids and Phenolic Compounds from Rosmarinus officinalis. J. Agric. Food Chem..

[20] Kuhlmann A., Röhl C. (2006). Phenolic Antioxidant Compounds Produced by in Vitro. Cultures of Rosemary (*Rosmarinus officinalis*) and Their Anti-inflammatory Effect on Lipopolysaccharide-Activated Microglia. Pharm. Biol..

[21] Visanji J.M., Thompson D.G., Padfield P.J. (2006). Induction of G2/M phase cell cycle arrest by carnosol and carnosic acid is associated with alteration of cyclin A and cyclin B1 levels. Cancer Lett..

[22] González-Vallinas M., Molina S., Vicente G., Zarza V., Martín-Hernández R., García-Risco M.R., Fornari T., Reglero G., De Molina A.R. (2014). Expression of MicroRNA-15b and the Glycosyltransferase GCNT3 Correlates with Antitumor Efficacy of Rosemary Diterpenes in Colon and Pancreatic Cancer. PLoS ONE.

[23] Xiang Q., Ma Y., Dong J., Shen R. (2015). Carnosic acid induces apoptosis associated with mitochondrial dysfunction and Akt inactivation in HepG2 cells. Int. J. Food Sci. Nutr..

[24] Kar S., Palit S., Ball W.B., Das P.K. (2012). Carnosic acid modulates Akt/IKK/NF-κB signaling by PP2A and induces intrinsic and extrinsic pathway mediated apoptosis in human prostate carcinoma PC-3 cells. Apoptosis.

[25] Barni M.V., Carlini M.J., Cafferata E.G., Puricelli L., Moreno S. (2012). Carnosic acid inhibits the proliferation and migration capacity of human colorectal cancer cells. Oncol. Rep..

[26] Aliebrahimi S., Kouhsari S.M., Arab S.S., Shadboorestan A., Ostad S.N. (2018). Phytochemicals, withaferin A and carnosol, overcome pancreatic cancer stem cells as c-Met inhibitors. Biomed. Pharmacother..

[27] Lo A.-H., Liang Y.-C., Lin-Shiau S.-Y., Ho C.-T., Lin J.-K. (2002). Carnosol, an antioxidant in rosemary, suppresses inducible nitric oxide synthase through down-regulating nuclear factor-κB in mouse macrophages. Carcinogenesis.

[28] Cheng A.-C., Lee M.-F., Tsai M.-L., Lai C.-S., Lee J.H., Ho C.-T., Pan M.-H. (2011). Rosmanol potently induces apoptosis through both the mitochondrial apoptotic pathway and death receptor pathway in human colon adenocarcinoma COLO 205 cells. Food Chem. Toxicol..

[29] Machado D.G., Cunha M.P., Neis V.B., Balen G.O., Colla A., Bettio L.E.B., Oliveira Á., Pazini F.L., Dalmarco J.B., Simionatto E.L. (2013). Antidepressant-like effects of fractions, essential oil, carnosol and betulinic acid isolated from Rosmarinus officinalis L.. Food Chem..

[30] Sasaki K., El Omri A., Kondo S., Han J., Isoda H. (2013). *Rosmarinus officinalis* polyphenols produce anti-depressant like effect through monoaminergic and cholinergic functions modulation. Behav. Brain Res..

[31] Romo Vaquero M., Garcia Villalba R., Larrosa M., Yáñez-Gascón M.J., Fromentin E., Flanagan J., Roller M., Tomás-Barberán F.A., Espín J.C., García-Conesa M.-T. (2013). Bioavailability of the major bioactive diterpenoids in a rosemary extract: Metabolic profile in the intestine, liver, plasma, and brain of Zucker rats. Mol. Nutr. Food Res..

[32] de Oliveira M.R. (2016). The dietary components carnosic acid and carnosol as neuroprotective agents: A mechanistic view. Mol. Neurobiol..

[33] Rasoolijazi H., Azad N., Joghataei M., Kerdari M., Nikbakht F., Soleimani M. (2013). The protective role of carnosic acid against beta-amyloid toxicity in rats. Sci. World J..

[34] Petersen M. (2013). Rosmarinic acid: New aspects. Phytochem. Rev..

[35] De Souza Gil E., Adrian Enache T., Maria Oliveira-Brett A. (2013). Redox behaviour of verbascoside and rosmarinic acid. Comb. Chem. High Throughput Screen..

[36] Fadel O., El Kirat K., Morandat S. (2011). The natural antioxidant rosmarinic acid spontaneously penetrates membranes to inhibit lipid peroxidation in situ. Biochim. Biophys. Acta.

[37] Kimura Y., Okuda H., Okuda T., Hatano T., Arichi S. (1987). Studies on the activities of tannins and related compounds, X. Effects of caffeetannins and related compounds on arachidonate metabolism in human polymorphonuclear leukocytes. J. Nat. Prod..

[38] Lucarini R., Bernardes W.A., Ferreira D.S., Tozatti M.G., Furtado R., Bastos J.K., Pauletti P.M., Januário A.H., Silva M.L.A., Cunha W.R. (2013). In vivo analgesic and anti-inflammatory activities of *Rosmarinus officinalis* aqueous extracts, rosmarinic acid and its acetyl ester derivative. Pharm. Biol..

[39] Amaral G.P., Mizdal C.R., Stefanello S.T., Mendez A.S.L., Puntel R.L., de Campos M.M.A., Soares F.A.A., Fachinetto R. (2019). Antibacterial and antioxidant effects of *Rosmarinus officinalis* L. extract and its fractions. J. Tradit. Complement. Med..

[40] Radziejewska I., Supruniuk K., Nazaruk J., Karna E., Poplawska B., Bielawska A., Galicka A. (2018). Rosmarinic acid influences collagen, MMPs, TIMPs, glycosylation and MUC1 in CRL-1739 gastric cancer cell line. Biomed. Pharmacother..

[41] Ma Z.-J., Yan H., Wang Y.-J., Yang Y., Li X.-B., Shi A.-C., Jing-Wen X., Yu-Bao L., Li L., Wang X.-X. (2018). Proteomics analysis demonstrating rosmarinic acid suppresses cell growth by blocking the glycolytic pathway in human HepG2 cells. Biomed. Pharmacother..

[42] Cui H.-Y., Zhang X.-J., Yang Y., Zhang C., Zhu C.-H., Miao J.-Y., Chen R. (2018). Rosmarinic acid elicits neuroprotection in ischemic stroke via Nrf2 and heme oxygenase 1 signaling. Neural Regen. Res..

[43] Wang J., Li G., Rui T., Kang A., Li G., Fu T., Li J., Di L., Cai B. (2017). Pharmacokinetics of rosmarinic acid in rats by LC-MS/MS: Absolute bioavailability and dose proportionality. RSC Adv..

[44] da Silva S.B., Amorim M., Fonte P., Madureira R., Ferreira D., Pintado M., Sarmento B. (2015). Natural extracts into chitosan nanocarriers for rosmarinic acid drug delivery. Pharm. Biol..

[45] Madureira A.R., Campos D.A., Fonte P., Nunes S., Reis F., Gomes A.M., Sarmento B., Pintado M.M. (2015). Characterization of solid lipid nanoparticles produced with carnauba wax for rosmarinic acid oral delivery. RSC Adv..

[46] Xu X., Huang M., Zou X. (2018). Docking-based inverse virtual screening: Methods, applications, and challenges. Biochem. Biophys. Rep..

[47] Warrier S.B., Kharkar P.S. (2016). Inverse Virtual Screening in Drug Repositioning: Detailed Investigation and Case Studies. Crystallizing Ideas–The Role of Chemistry.

[48] Konc J. (2019). Identification of neurological disease targets of natural products by computational screening. Neural Regen. Res..

[49] Kores K., Lešnik S., Bren U., Janežič D., Konc J. (2019). Discovery of novel potential human targets of resveratrol by inverse molecular docking. J. Chem. Inf. Model..

[50] Furlan V., Konc J., Bren U. (2018). Inverse molecular docking as a novel approach to study anticarcinogenic and anti-neuroinflammatory effects of curcumin. Molecules.

[51] Sterling T., Irwin J.J. (2015). ZINC 15–ligand discovery for everyone. J. Chem. Inf. Model..

[52] Frisch M., Trucks G., Schlegel H., Scuseria G., Robb M., Cheeseman J., Scalmani G., Barone V., Petersson G., Nakatsuji H. (2016). Gaussian 16.

[53] Duraán-Iturbide N.A., Díaz-Eufracio B.r.I., Medina-Franco J.L. (2020). In silico ADME/Tox profiling of natural products: A focus on BIOFACQUIM. ACS Omega.

[54] Daina A., Michielin O., Zoete V. (2017). SwissADME: A free web tool to evaluate pharmacokinetics, drug-likeness and medicinal chemistry friendliness of small molecules. Sci. Rep..

[55] Fine J., Konc J., Samudrala R., Chopra G. (2020). Candock: Chemical atomic network-based hierarchical flexible docking algorithm using generalized statistical potentials. J. Chem. Inf. Model..

[56] Konc J., Lešnik S., Škrlj B., Janežič D. (2021). ProBiS-Dock Database: A Web Server and Interactive Web Repository of Small Ligand–Protein Binding Sites for Drug Design. J. Chem. Inf. Model..

[57] Konc J., Janežič D. (2007). An improved branch and bound algorithm for the maximum clique problem. MATCH Commun. Math. Comput. Chem..

[58] Konc J., Miller B.T., Štular T., Lešnik S., Woodcock H.L., Brooks B.R., Janežič D. (2015). ProBiS-CHARMMing: Web interface for prediction and optimization of ligands in protein binding sites. J. Chem. Inf. Model..

[59] Triballeau N., Acher F., Brabet I., Pin J.-P., Bertrand H.-O. (2005). Virtual screening workflow development guided by the “receiver operating characteristic” curve approach. Application to high-throughput docking on metabotropic glutamate receptor subtype 4. J. Med. Chem..

[60] Truchon J.-F., Bayly C.I. (2007). Evaluating virtual screening methods: Good and bad metrics for the “early recognition” problem. J. Chem. Inf. Model..

[61] Empereur-Mot C., Guillemain H., Latouche A., Zagury J.-F., Viallon V., Montes M. (2015). Predictiveness curves in virtual screening. J. Cheminform..

[62] Gaulton A., Bellis L.J., Bento A.P., Chambers J., Davies M., Hersey A., Light Y., McGlinchey S., Michalovich D., Al-Lazikani B. (2012). ChEMBL: A large-scale bioactivity database for drug discovery. Nucleic Acids Res..

[63] Sheridan R.P., Singh S.B., Fluder E.M., Kearsley S.K. (2001). Protocols for bridging the peptide to nonpeptide gap in topological similarity searches. J. Chem. Inf. Comput. Sci..

[64] Empereur-Mot C., Zagury J.-F., Montes M. (2016). Screening explorer–An interactive tool for the analysis of screening results. J. Chem. Inf. Model..

[65] Ahmad H.H., Hamza A.H., Hassan A.Z., Sayed A.H. (2013). Promising therapeutic role of Rosmarinus officinalis successive methanolic fraction against colorectal cancer. Int. J. Pharm. Pharm. Sci.

[66] Sacco C., Bellumori M., Santomauro F., Donato R., Capei R., Innocenti M., Mulinacci N. (2015). An in vitro evaluation of the antibacterial activity of the non-volatile phenolic fraction from rosemary leaves. Nat. Prod. Res..

[67] Moreno S., Scheyer T., Romano C.S., Vojnov A.A. (2006). Antioxidant and antimicrobial activities of rosemary extracts linked to their polyphenol composition. Free Radic. Res..

[68] Pavić V., Jakovljević M., Molnar M., Jokić S. (2019). Extraction of carnosic acid and carnosol from sage (*Salvia officinalis* L.) leaves by supercritical fluid extraction and their antioxidant and antibacterial activity. Plants.

[69] Pariš A., Štrukelj B., Renko M., Turk V., Pukl M., Umek A., Korant B.D. (1993). Inhibitory Effect of Carnosolic Acid on HIV-1 Protease in Cell-Free Assays. J. Nat. Prod..

[70] Falo M., Fillmore H., Reeves T., Phillips L. (2006). Matrix metalloproteinase-3 expression profile differentiates adaptive and maladaptive synaptic plasticity induced by traumatic brain injury. J. Neurosci. Res..

[71] López-Jiménez A., García-Caballero M., Medina M.Á., Quesada A.R. (2013). Anti-angiogenic properties of carnosol and carnosic acid, two major dietary compounds from rosemary. Eur. J. Nutr..

[72] Kranenburg O. (2005). The KRAS oncogene: Past, present, and future. Biochim. Biophys. Acta.

[73] Matikas A., Mistriotis D., Georgoulias V., Kotsakis A. (2017). Targeting KRAS mutated non-small cell lung cancer: A history of failures and a future of hope for a diverse entity. Crit. Rev. Oncol. Hematol..

[74] Fell J.B., Fischer J.P., Baer B.R., Blake J.F., Bouhana K., Briere D.M., Brown K.D., Burgess L.E., Burns A.C., Burkard M.R. (2020). Identification of the clinical development candidate MRTX849, a covalent KRASG12C inhibitor for the treatment of cancer. J. Med. Chem..

[75] Ahmed Z., Abdeslam-Hassan M., Ouassila L., Danielle B. (2012). Extraction and modeling of Algerian rosemary essential oil using supercritical CO_2_: Effect of pressure and temperature. Energy Procedia.

[76] Balskus E.P. (2015). Colibactin: Understanding an elusive gut bacterial genotoxin. Nat. Prod. Rep..

[77] Teplyakov A., Obmolova G., Badet-Denisot M.-A., Badet B., Polikarpov I. (1998). Involvement of the C terminus in intramolecular nitrogen channeling in glucosamine 6-phosphate synthase: Evidence from a 1.6\AA crystal structure of the isomerase domain. Structure.

[78] Bearne S.L., Blouin C. (2000). Inhibition of Escherichia coli Glucosamine-6-phosphate Synthase by Reactive Intermediate Analogues: The Role of the 2-amino function in Catalysis. J. Biol. Chem..

[79] Fadaka A., Ajiboye B., Ojo O., Adewale O., Olayide I., Emuowhochere R. (2017). Biology of glucose metabolization in cancer cells. J. Oncol. Sci..

[80] Vander Heiden M.G., Christofk H.R., Schuman E., Subtelny A.O., Sharfi H., Harlow E.E., Xian J., Cantley L.C. (2010). Identification of small molecule inhibitors of pyruvate kinase M2. Biochem. Pharmacol..

[81] Skehel J.J., Wiley D.C. (2000). Receptor binding and membrane fusion in virus entry: The influenza hemagglutinin. Annu. Rev. Biochem..

[82] Wong S.-S., Webby R.J. (2013). Traditional and new influenza vaccines. Clin. Microbiol. Rev..

[83] Kadam R.U., Wilson I.A. (2017). Structural basis of influenza virus fusion inhibition by the antiviral drug Arbidol. Proc. Natl. Acad. Sci. USA.

[84] Lv Z., Chu Y., Wang Y. (2015). HIV protease inhibitors: A review of molecular selectivity and toxicity. HIV AIDS.

[85] Malhotra S., Dhundial R., Bhatia N., Duggal N. (2018). HIV-2 Infections from a Tertiary Care Hospital in India-A Case Report. J. Hum. Virol. Retrovirol..

[86] De Silva T., Weiss R.A. (2010). HIV-2 goes global: An unaddressed issue in Indian anti-retroviral programmes. Indian J. Med. Res..

[87] Visseaux B., Damond F., Matheron S., Descamps D., Charpentier C. (2016). HIV-2 molecular epidemiology. Infect. Genet. Evol..

[88] De Silva T.I., van Tienen C., Rowland-Jones S.L., Cotten M. (2010). Dual infection with HIV-1 and HIV-2: Double trouble or destructive interference?. HIV Ther..

[89] Annabel B., Anna D., Hannah M. (2019). Global Tuberculosis Report 2019.

[90] Garzan A., Willby M.J., Green K.D., Tsodikov O.V., Posey J.E., Garneau-Tsodikova S. (2016). Discovery and optimization of two Eis inhibitor families as kanamycin adjuvants against drug-resistant M. tuberculosis. ACS Med. Chem. Lett..

[91] Houghton J.L., Biswas T., Chen W., Tsodikov O.V., Garneau-Tsodikova S. (2013). Chemical and structural insights into the regioversatility of the aminoglycoside acetyltransferase Eis. Chembiochem.

[92] Wu C.-C., Baiga T.J., Downes M., La Clair J.J., Atkins A.R., Richard S.B., Fan W., Stockley-Noel T.A., Bowman M.E., Noel J.P. (2017). Structural basis for specific ligation of the peroxisome proliferator-activated receptor δ. Proc. Natl. Acad. Sci. USA.

[93] Dressel U., Allen T.L., Pippal J.B., Rohde P.R., Lau P., Muscat G.E. (2003). The peroxisome proliferator-activated receptor β/δ agonist, GW501516, regulates the expression of genes involved in lipid catabolism and energy uncoupling in skeletal muscle cells. Mol. Endocrinol..

[94] Liu Y., Colby J.K., Zuo X., Jaoude J., Wei D., Shureiqi I. (2018). The role of PPAR-δ in metabolism, inflammation, and cancer: Many characters of a critical transcription factor. Int. J. Mol. Sci..

[95] Waku T., Shiraki T., Oyama T., Maebara K., Nakamori R., Morikawa K. (2010). The nuclear receptor PPARγ individually responds to serotonin-and fatty acid-metabolites. EMBO J..

[96] Alexacou K.-M., Tenchiu A.-C., Chrysina E.D., Charavgi M.-D., Kostas I.D., Zographos S.E., Oikonomakos N.G., Leonidas D.D. (2010). The binding of β-d-glucopyranosyl-thiosemicarbazone derivatives to glycogen phosphorylase: A new class of inhibitors. Biorg. Med. Chem..

[97] Treadway J.L., Mendys P., Hoover D.J. (2001). Glycogen phosphorylase inhibitors for treatment of type 2 diabetes mellitus. Expert Opin. Investig. Drugs.

[98] Spasov A., Chepljaeva N., Vorob’ev E. (2016). Glycogen phosphorylase inhibitors in the regulation of carbohydrate metabolism in type 2 diabetes. Russ. J. Bioorganic Chem..

[99] Martin J.L., Johnson L.N., Withers S.G. (1990). Comparison of the binding of glucose and glucose 1-phosphate derivatives to T-state glycogen phosphorylase b. Biochemistry.

[100] Barbier P., Tsvetkov P.O., Breuzard G., Devred F. (2014). Deciphering the molecular mechanisms of anti-tubulin plant derived drugs. Phytochem. Rev..

[101] Nawrotek A., Knossow M., Gigant B. (2011). The determinants that govern microtubule assembly from the atomic structure of GTP-tubulin. J. Mol. Biol..

[102] Bomalaski J.S., Clark M.A. (1993). Phospholipase A2 and arthritis. Arthritis Rheum..

[103] Cummings B.S. (2007). Phospholipase A2 as targets for anti-cancer drugs. Biochem. Pharmacol..

[104] Mallat Z., Lambeau G., Tedgui A. (2010). Lipoprotein-associated and secreted phospholipases A2 in cardiovascular disease: Roles as biological effectors and biomarkers. Circulation.

[105] Ong W.-Y., Farooqui T., Kokotos G., Farooqui A.A. (2015). Synthetic and natural inhibitors of phospholipases A2: Their importance for understanding and treatment of neurological disorders. ACS Chem. Neurosci..

[106] Ivy S.P., Wick J.Y., Kaufman B.M. (2009). An overview of small-molecule inhibitors of VEGFR signaling. Nat. Rev. Clin. Oncol..

[107] Kufareva I., Abagyan R. (2008). Type-II kinase inhibitor docking, screening, and profiling using modified structures of active kinase states. J. Med. Chem..

[108] Vásquez A.F., Reyes Munoz A., Duitama J., González Barrios A. (2021). Discovery of new potential CDK2/VEGFR2 type II inhibitors by fragmentation and virtual screening of natural products. J. Biomol. Struct. Dyn..

[109] Rathi E., Kumar A., Kini S.G. (2019). Molecular dynamics guided insight, binding free energy calculations and pharmacophore-based virtual screening for the identification of potential VEGFR2 inhibitors. J. Recept. Signal Transduct..

[110] Banerjee A.K., Arora N., Murty U.S.N. (2012). Aspartate carbamoyltransferase of *Plasmodium falciparum* as a potential drug target for designing anti-malarial chemotherapeutic agents. Med. Chem. Res..

[111] Bosch S.S., Lunev S., Batista F.A., Linzke M., Kronenberger T., Dömling A.S., Groves M.R., Wrenger C. (2020). Molecular Target Validation of Aspartate Transcarbamoylase from *Plasmodium falciparum* by Torin 2. ACS Infect. Dis..

[112] Wang J., Stieglitz K.A., Cardia J.P., Kantrowitz E.R. (2005). Structural basis for ordered substrate binding and cooperativity in aspartate transcarbamoylase. Proc. Natl. Acad. Sci. USA.

[113] Dos Santos J.I., Cardoso F.F., Soares A.M., dal Pai Silva M., Gallacci M., Fontes M.R. (2011). Structural and functional studies of a bothropic myotoxin complexed to rosmarinic acid: New insights into Lys49-PLA2 inhibition. PLoS ONE.

[114] Han S., Li X., Xia Y., Yu Z., Cai N., Malwal S.R., Han X., Oldfield E., Zhang Y. (2019). Farnesyl Pyrophosphate Synthase as a Target for Drug Development: Discovery of Natural-Product-Derived Inhibitors and Their Activity in Pancreatic Cancer Cells. J. Med. Chem..

[115] Hoffman M., Monroe D., Oliver J., Roberts H. (1995). Factors IXa and Xa play distinct roles in tissue factor-dependent initiation of coagulation. Blood.

[116] Chen X., Zhou L., Zhang Y., Yi D., Liu L., Rao W., Wu Y., Ma D., Liu X., Zhou X.-H.A. (2014). Risk factors of stroke in Western and Asian countries: A systematic review and meta-analysis of prospective cohort studies. BMC Public Health.

[117] Li S., Peng Y., Wang X., Qian Y., Xiang P., Wade S.W., Guo H., Lopez J.A.G., Herzog C.A., Handelsman Y. (2019). Cardiovascular events and death after myocardial infarction or ischemic stroke in an older Medicare population. Clin. Cardiol..

[118] Perzborn E., Roehrig S., Straub A., Kubitza D., Mueck W., Laux V. (2010). Rivaroxaban: A new oral factor Xa inhibitor. Atertio. Thromb. Vasc. Biol..

[119] Komoriya S., Kobayashi S., Osanai K., Yoshino T., Nagata T., Haginoya N., Nakamoto Y., Mochizuki A., Nagahara T., Suzuki M. (2006). Design, synthesis, and biological activity of novel factor Xa inhibitors: Improving metabolic stability by S1 and S4 ligand modification. Biorg. Med. Chem..

[120] Alcaraz L.A., Banci L., Bertini I., Cantini F., Donaire A., Gonnelli L. (2007). Matrix metalloproteinase–inhibitor interaction: The solution structure of the catalytic domain of human matrix metalloproteinase-3 with different inhibitors. J. Biol. Inorg. Chem..

[121] World Helath Organization (2010). Control of the Leishmaniases WHO Technical Report Series 949.

[122] Martin M.B., Grimley J.S., Lewis J.C., Heath H.T., Bailey B.N., Kendrick H., Yardley V., Caldera A., Lira R., Urbina J.A. (2001). Bisphosphonates Inhibit the Growth of Trypanosoma b rucei, Trypanosoma c ruzi, Leishmania d onovani, Toxoplasma g ondii, and Plasmodium f alciparum: A Potential Route to Chemotherapy. J. Med. Chem..

[123] Jin L., Li D., Alesi G.N., Fan J., Kang H.-B., Lu Z., Boggon T.J., Jin P., Yi H., Wright E.R. (2015). Glutamate dehydrogenase 1 signals through antioxidant glutathione peroxidase 1 to regulate redox homeostasis and tumor growth. Cancer Cell.

[124] McDermott L.A., Iyer P., Vernetti L., Rimer S., Sun J., Boby M., Yang T., Fioravanti M., O’Neill J., Wang L. (2016). Design and evaluation of novel glutaminase inhibitors. Biorg. Med. Chem..

[125] Li C., Allen A., Kwagh J., Doliba N.M., Qin W., Najafi H., Collins H.W., Matschinsky F.M., Stanley C.A., Smith T.J. (2006). Green tea polyphenols modulate insulin secretion by inhibiting glutamate dehydrogenase. J. Biol. Chem..

[126] Li C., Li M., Chen P., Narayan S., Matschinsky F.M., Bennett M.J., Stanley C.A., Smith T.J. (2011). Green tea polyphenols control dysregulated glutamate dehydrogenase in transgenic mice by hijacking the ADP activation site. J. Biol. Chem..

[127] Li M., Smith C.J., Walker M.T., Smith T.J. (2009). Novel Inhibitors Complexed with Glutamate Dehydrogenase Allosteric Regulation by Control of Protein Dynamics. J. Biol. Chem..

[128] DeLaBarre B., Gross S., Fang C., Gao Y., Jha A., Jiang F., Song J.J., Wei W., Hurov J.B. (2011). Full-length human glutaminase in complex with an allosteric inhibitor. Biochemistry.

[129] Thangavelu K., Pan C.Q., Karlberg T., Balaji G., Uttamchandani M., Suresh V., Schüler H., Low B.C., Sivaraman J. (2012). Structural basis for the allosteric inhibitory mechanism of human kidney-type glutaminase (KGA) and its regulation by Raf-Mek-Erk signaling in cancer cell metabolism. Proc. Natl. Acad. Sci. USA.

[130] Furlan V., Bren U. (2021). Insight into Inhibitory Mechanism of PDE4D by Dietary Polyphenols Using Molecular Dynamics Simulations and Free Energy Calculations. Biomolecules.

[131] Kores K., Konc J., Bren U. (2021). Mechanistic insights into side effects of troglitazone and rosiglitazone using a novel inverse molecular docking protocol. Pharmaceutics.

[132] Jukič M., Kores K., Janežič D., Bren U. (2021). Repurposing of Drugs for SARS-CoV-2 Using Inverse Docking Fingerprints. Front. Chem..

