# Supplementary material for: Mechanistic Insights into Biological Activities of Polyphenolic Compounds from Rosemary Obtained by Inverse Molecular Docking
Source: Foods. 2021 Dec 28;11(1):67. doi: 10.3390/foods11010067 (PMC8750736; doi:10.3390/foods11010067)
Supplement: Supplementary file 1 [file foods-11-00067-s001.zip › SI.pdf]

foods

MDPI

Supplementary Materials: Mechanistic Insights into Biological Activities of Polyphenolic Compounds from Rosemary Obtained by Inverse Molecular Docking

Samo Lešnik and Urban Bren

Table S1. Best docking scores of ligands/drugs known to bind to a specific target.

PDB ID with chain	Ligand ID	Predicted ligand docking score (arb. units)
4lucB	M1X	−79,52
3oojA	G6P	−50,66
1kenA	75U	−47,43
4jd6C	TOY	−68,64
3mt7A	16O	−58,29
3rycC	LOC	−41,68
2p2hA	STI	−50,02
2d1jA	D01	−58,50
2jt5A	JT5	−66,79
4jzbA	P2H	−51,53
3qmuB	B1T	−35,91
5fi6A	04A	−61,02

Table S2. Best docking scores for carnosol, carnosic acid and rosmanol.

PDB ID with chain	Ligand	Predicted ligand docking score (arb. units)	Protein name	Organism
5ilhA	carnosol	−70.6	5-epi-aristolochene synthase	*Nicotiana tabacum*
4lucB*	carnosic acid	−69.9	K-Ras G12C	*Homo sapiens*
1g0hA	carnosol	−69.0	Inositol monophosphatase	*Methanocaldococcus jannaschii*
5ilhA	rosmanol	−68.5	5-epi-aristolochene synthase	*Nicotiana tabacum*
3oojA*	carnosic acid	−68.2	Glucosamine-fructose-6-phosphate aminotransferase	*Escherichia coli*
3srdD*	carnosic acid	−68.1	Pyruvate kinase M2	*Homo sapiens*
3bjtC	carnosic acid	−67.6	Pyruvate kinase M2	*Homo sapiens*
2w27A	rosmanol	−66.9	YKUI protein	*Bacillus subtilis*
1kenA*	carnosic acid	−66.9	Hemagglutinin HA1	*Influenza A virus*
4qg8D	carnosic acid	−66.8	Pyruvate kinase M2	*Homo sapiens*
3oyrB	rosmanol	−66.7	Trans-isoprenyl diphosphate synthase	*Caulobacter vibrioides*
4lv6B	carnosic acid	−65.7	K-Ras G12C	*Homo sapiens*
4lltB	carnosic acid	−65.6	Geranyltranstransferase	*Roseobacter denitrificans*
1pkmA	carnosic acid	−65.3	Pyruvate kinase M2	*Felis catus*
3srfE	carnosic acid	−65.1	Pyruvate kinase M2	*Homo sapiens*
3u2zA	carnosic acid	−65.0	Pyruvate kinase M2	*Homo sapiens*
2hpeA*	carnosic acid	−65.0	HIV-2 protease	*Human immunodeficiency virus 2*

2uulK	carnosic acid	−65.0	C-phycocyanin	*Phormidium sp.*
5v6vA	carnosic acid	−64.9	K-Ras G12C	*Homo sapiens*
4jd6C*	carnosic acid	−64.8	Enhanced intracellular survival protein	*Mycobacterium tuberculosis*
5u46A*	carnosic acid	−64.7	Peroxisome proliferator activated receptor delta	*Homo sapiens*
4ffbA	carnosic acid	−64.5	Tubulin	*Saccharomyces cerevisiae*
3mt7A*	carnosic acid	−64.5	Glycogen phosphorylase	*Oryctolagus cuniculus*
5x1vA	carnosic acid	−64.2	Pyruvate kinase M2	*Homo sapiens*
5x1vB	carnosic acid	−64.2	Pyruvate kinase M2	*Homo sapiens*
3rycC*	carnosic acid	−64.2	Tubulin	*Rattus norvegicus*
5x1vD	carnosic acid	−64.1	Pyruvate kinase M2	*Homo sapiens*
4o4lA	carnosic acid	−64.0	Tubulin	*Bos taurus*
5v0tA	carnosic acid	−63.9	Trehalose-phosphate synthase	*Paraburkholderia xenovorans*
4wj8A	carnosic acid	−63.9	Pyruvate kinase M2	*Homo sapiens*
4rppB	carnosic acid	−63.9	Pyruvate kinase M2	*Homo sapiens*
3cdxE	rosmanol	−63.7	Succinylglutamatedesuccinylase/aspartoacylase	*Cereibacter sphaeroides*
3cdxE	carnosic acid	−63.6	Succinylglutamatedesuccinylase/aspartoacylase	*Cereibacter sphaeroides*
4o4iA	carnosic acid	−63.6	Tubulin	*Bos taurus*
3bjtB	carnosic acid	−63.5	Pyruvate kinase M2	*Homo sapiens*
2ps1A	carnosic acid	−63.5	Orotate phosphoribosyltransferase 1	*Saccharomyces cerevisiae*
2j9kB	carnosic acid	−63.5	HIV-1 protease	*Human immunodeficiency virus 1*
1fxfB*	carnosol	−63.3	Phospholipase A2	*Sus scrofa*
3ogpA*	carnosic acid	−63.3	FIV protease	*Felis catus*
4i50C	carnosic acid	−63.2	Tubulin	*Bos taurus*
3h6oA	carnosic acid	−63.1	Pyruvate kinase M2	*Homo sapiens*
2p2hA*	carnosic acid	−63.1	Vascular endothelial growth factor receptor 2	*Homo sapiens*
5ilqC*	carnosic acid	−63.0	Aspartate carbamoyltransferase	*Plasmodium falciparum*
3h6oC	carnosic acid	−63.0	Pyruvate kinase M2	*Homo sapiens*
5jqgA	carnosic acid	−63.0	Tubulin	*Sus scrofa*
3h6oB	carnosic acid	−63.0	Pyruvate kinase M2	*Homo sapiens*
4iv5A*	carnosic acid	−62.8	Aspartate carbamoyltransferase	*Trypanosoma cruzi*
1mrxB	carnosic acid	−62.8	Hiv-1 protease	*Human immunodeficiency virus 1*
3fpfA	carnosic acid	−62.8	Uncharacterized protein	*Methanothermobacter thermautotrophicus*

* Is described in the main text.

Table S3. Best docking scores for rosmarinic acid.

PDB ID with chain	Predicted ligand docking score (arb. units)	Protein name	Organism
2d1jA*	−86.1	Factor Xa	*Homo sapiens*
1fxfB*	−84.8	Phospholipase A2	*Sus scrofa*
2jt5A*	−84.5	Matrix metalloproteinase 3	*Homo sapiens*
4jzbA*	−83.2	Farnesyl pyrophosphate synthase	*Leishmania major*
2w27A	−80.9	YKUI protein	*Bacillus subtilis*

3qmuB*	−80.2	Glutamate dehydrogenase 1	*Bos taurus*
3qxgB	−79.3	Inorganic pyrophosphatase	*Bacteroides thetaiotaomicron*
4jzxA	−79.1	Farnesyl pyrophosphate synthase	*Leishmania major*
3m1rC	−78.8	Formimidoylglutamase	*Bacillus subtilis*
5fi6A*	−78.6	Glutaminase	*Homo sapiens*

* Is described in the main text.

Table S4. Interactions of carnosic acid with K-Ras.

Contact residue	Closest contact distance (A)
Hydrophobic interactions	
Glu62-A	3.81
Tyr96-A	3.29
Gln99-A	2.74
Hydrogen bonds	
Thr58-A	3.45
Ala59-A	3.86
Gly60-A	3.52
Salt bridge	
Arg68-A	4.10

Table S5. Interactions of carnosic acid with glucosamine/fructose-6-phosphate aminotransferase.

Contact residue	Closest contact distance (A)
Hydrophobic interactions	
Val399-A	3.87
Leu601-A	3.69
Val605-A	3.92
Hydrogen bonds	
Thr302-A	3.01
Ser303-A	3.23
Ser401-A	2.73
Salt bridge	
Lys603-A	2.96

Table S6. Interactions of carnosic acid with Pyruvate kinase 2 – muscle isoform.

Contact residue	Closest contact distance (A)
Hydrophobic interactions	
Phe-26-B	3.78
Asn-350-B	3.90
Leu-353-B	3.55
Leu353-D	3.84
Ile389-D	3.79
Tyr390-D	3.72
Leu394-D	3.71
Hydrogen bonds	
Asn350-B	3.38
Asp354-D	2.49
Salt bridge	

Lys311-D	3.42
π-stacking	
Phe26-B	5.10

Table S7. Interactions of carnosic acid with Hemagglutinin HA1.

Contact residue	Closest contact distance (A)
Hydrophobic interactions	
Val55-D	3.92
Trp92-D	3.69
Glu97-B	3.73
Leu99-D	3.70
Pro293-C	3.53
Phe294-C	3.52
Lys307-C	3.96
Hydrogen bonds	
Thr59-D	3.33
Lys307-C	2.86
Lys310-A	2.91

Table S8. Interactions of carnosic acid with HIV-2 protease.

Contact residue	Closest contact distance (A)
Hydrophobic interactions	
Ala28-A	3.63
Ala28-B	3.98
Ile32-A	3.77
Val47-A	3.71
Hydrogen bonds	
Ile50-A	3.20

Table S9. Interactions of carnosic acid with enhanced intra-cellular survival protein.

Contact residue	Closest contact distance (A)
Hydrophobic interactions	
Phe24-C	3.83
Ile28-C	3.74
Trp36-C	3.85
Phe84-C	3.61
Val85-C	3.92
Leu118-C	3.76
Hydrogen bonds	
His119-C	3.05

Table S10. Interactions of carnosic acid with Peroxisome proliferator-activated receptor δ.

Contact residue	Closest contact distance (A)
Hydrophobic interactions	
Phe190-A	3.83
Met192-A	3.74
Thr252-A	3.90
Glu255-A	3.94

Met293-A	3.77
Ile297-A	3.71
Hydrogen bonds	
Asn191-A	2.98
Met192-A	2.88
Glu259-A	3.20

Table S11. Interactions of carnosic acid glycogen phosphorylase.

Contact residue	Closest contact distance (A)
Hydrophobic interactions	
Leu139-A	3.61
Asp283-A	3.53
Val455-A	3.25
Arg569-A	3.80
Hydrogen bonds	
Lys574-A	2.75
Thr676-A	4.10

Table S12. Interactions of carnosic acid with tubulin.

Contact residue	Closest contact distance (A)
Hydrophobic interactions	
Gln247-D	3.80
Thr325-D	3.98
Hydrogen bonds	
Pro175-C	2.73
Ser178-C	3.59

Table S13. Interactions of carnosic acid with HIV-1 protease.

Contact residue	Closest contact distance (A)
Hydrophobic interactions	
Ala28-B	3.78
Ile48-B	3.53
Ile50-A	3.95
Hydrogen bonds	
Asp25-A	4.04
Asp29-B	3.18
Salt bridge	
Arg8-A	3.99

Table S14. Interactions of carnosol with Phospholipase A2.

Contact residue	Closest contact distance (A)
Hydrophobic interactions	
Leu18-A	3.79
Leu2-B	3.24
Phe5-B	3.73
Arg6-B	3.90
Pro18-B	3.17
Leu19-B	3.56

Phe22-B	3.95
Leu31-B	3.78
Tyr69-B	3.77
Hydrogen bonds	
Tyr69-B	2.89

Table S15. Interactions of carnosic acid with FIV protease.

Contact residue	Closest contact distance (A)
Hydrophobic interactions	
Ile57-B	3.82
Ile59-B	3.73
Hydrogen bonds	
Asp29-B	2.80

Table S16. Interactions of carnosic acid vascular endothelial growth factor receptor 2

Contact residue	Closest contact distance (A)
Hydrophobic interactions	
Ile1025-A	2.94
His1026-A	2.74
Asp1028-A	3.57
Leu1049-A	3.08
Ala1050-A	3.42
Salt bridges	
Arg1027-A	4.37

Table S17. Interactions of carnosic acid with aspartate carbamoyltransferase (P. falciparum).

Contact residue	Closest contact distance (A)
Hydrophobic interactions	
Lys138-A	3.99
Hydrogen bonds	
Ser107-C	3.08
Arg109-C	2.72
110-C	2.73
Salt bridges	
Arg159-C	3.15
His187-C	5.16
π-cation interaction	
Arg109-C	4.81

Table S18. Interactions of carnosic acid with aspartate carbamoyltransferase (T. cruzi).

Contact residue	Closest contact distance (A)
Hydrophobic interactions	
Arg64-A	3.96
Asp169-A	3.71
Thr175-A	3.71
Arg242-A	3.84
Pro288-A	3.62
Hydrogen bonds	

Arg113-A	2.85
Arg174-A	3.49
Salt bridges	
Lys92-B	5.00
Arg242-A	3.57

Table S19. Interactions of rosmarinic acid with coagulation factor X.

Contact residue	Closest contact distance (A)
Hydrophobic interactions	
Tyr99-A	3.62
Gln192-A	3.49
Trp215-A	3.37
Cys220-A	3.47
Tyr228-A	4.66
π-stacking	
Phe174-A	3.84

Table S20. Interactions of rosmarinic acid with phospholipase A2.

Contact residue	Closest contact distance (A)
Hydrophobic interactions	
Leu2-A	3.92
Phe5-A	3.90
Asn23-A	3.89
Leu31-A	3.97
Asp49-A	3.68
Tyr52-A	3.73
Tyr69-A	3.88
Hydrogen bonds	
Pro18-A	2.71
Gly30-A	2.97
Asp49-A	2.99
Arg53-A	3.49
Tyr-69-A	2.93
Salt bridges	
His48-A	3.61

Table S21. Interactions of rosmarinic acid with matrix metalloproteinase-3.

Contact residue	Closest contact distance (A)
Hydrophobic interactions	
Leu197-A	3.79
Tyr220-A	4.00
Tyr223-A	3.83
Hydrogen bonds	
Asn162-A	2.94
Ala165-A	3.19
Tyr223-A	2.92
Thr215-A	4.59

Table S22. Interactions of rosmarinic acid with farnesyl pyrophosphate synthase.

Contact residue	Closest contact distance (A)
Hydrophobic interactions	
Phe94-A	3.55
Leu95-A	3.62
Asp98-A	3.78
Thr163-A	3.44
Gln167-A	3.41
Lys207-A	3.74
Phe246-A	3.82
Hydrogen bonds	
Lys48-A	3.75
Arg51-A	3.84
Asn126-B	3.25
Gln247-A	2.24
Salt bridges	
Arg51-A	4.75
Lys207-A	4.83
π-stacking	
Phe94-A	4.25
π-cation interactions	
Lys264	4.53

Table S23. Interactions of rosmarinic acid with glutamate dehydrogenase 1.

Contact residue	Closest contact distance (A)
Hydrophobic interactions	
Ile65-B	3.81
Arg66-B	3.85
Lys143-B	3.96
Lys143-C	3.65
Arg146-C	3.80
Arg147-B	3.66
Phe500E	3.53
Hydrogen bonds	
Arg66-B	2.91
Glu142-C	2.74
Lys143-C	2.74
Arg146-C	3.07
Arg147-C	2.88
Thr501-E	2.70
π-stacking	
Phe500-E	4.13

Table S24. Interactions of rosmarinic acid with glutaminase.

Contact residue	Closest contact distance (A)
Hydrophobic interactions	
Lys319-A	3.60
Lys319-B	3.70
Leu320-A	3.53
Phe321-A	3.75
Leu322-A	3.81
Tyr393-A	3.47
Hydrogen bonds	
Phe321-B	2.77
Leu322-A	3.95
Leu322-B	2.80
Glu324-B	4.08
Asp326-A	3.56
Asp326-B	2.59
Tyr393-B	4.10
π-cation interactions	
Lys319-A	4.59
